# Functional Characterization of UDP-Glycosyltransferases Involved in Anti-viral Lignan Glycosides Biosynthesis in *Isatis indigotica*

**DOI:** 10.3389/fpls.2022.921815

**Published:** 2022-06-14

**Authors:** Yuping Tan, Jian Yang, Yinyin Jiang, Jian Wang, Yahui Liu, Yujun Zhao, Baolong Jin, Xing Wang, Tong Chen, Liping Kang, Juan Guo, Guanghong Cui, Jinfu Tang, Luqi Huang

**Affiliations:** ^1^School of Traditional Chinese Medicine, Shenyang Pharmaceutical University, Shenyang, China; ^2^State Key Laboratory of Dao-di Herbs, National Resource Center for Chinese Materia Medica, China Academy of Chinese Medical Sciences, Beijing, China; ^3^National Institute of Metrology, Beijing, China; ^4^School of Traditional Chinese Medicine, Capital Medical University, Beijing, China; ^5^Beijing Key Lab of TCM Collateral Disease Theory Research, Capital Medical University, Beijing, China

**Keywords:** *Isatis indigotica*, lignan biosynthesis, glycosyltransferases, transgenic hairy roots, clemastanin B, diversification

## Abstract

*Isatis indigotica* is a popular herbal medicine with its noticeable antiviral properties, which are primarily due to its lignan glycosides such as lariciresinol-4-*O*-β-D-glucoside and lariciresinol-4,4′-bis-*O*-β-D-glucosides (also called clemastanin B). UDP-glucose-dependent glycosyltransferases are the key enzymes involved in the biosynthesis of these antiviral metabolites. In this study, we systematically characterized the UGT72 family gene IiUGT1 and two UGT71B family genes, IiUGT4 and IiUGT71B5a, with similar enzymatic functions. Kinetic analysis showed that IiUGT4 was more efficient than IiUGT1 or IiUGT71B5a for the glycosylation of lariciresinol. Further knock-down and overexpression of these *IiUGTs* in *I. indigotica’s* hairy roots indicates that they play different roles *in planta*: *IiUGT71B5a* primarily participates in the biosynthesis of coniferin not pinoresinol diglucoside, and *IiUGT1* primarily participates in the biosynthesis of pinoresinol diglucoside, while *IiUGT4* is responsible for the glycosylation of lariciresinol and plays a dominant role in the biosynthesis of lariciresinol glycosides in *I. indigotica*. Analysis of the molecular docking and site-mutagenesis of IiUGT4 have found that key residues for its catalytic activity are H373, W376, E397, and that F151 could be associated with substrate preference. This study elucidates the biosynthetic route of anti-viral lignan glycosides in *I. indigotica*, and provides the foundation for the production of anti-viral lignan glycosides *via* synthetic biology under the heterologous model.

## Introduction

*Isatis indigotica* is a popular plant used in traditional Chinese medicine and is distributed and cultivated widely across China. In traditional Chinese medicine, the roots and leaves of *I. indigotica* are known as “Băn Lán Gēn” and “Dà Qīng Yè,” respectively (Miceli et al., 2017; Beijing, 2020). Previous studies have demonstrated that Băn Lán Gēn and Dà Qīng Yè have various pharmacological activities (Oberthür et al. (2004); Mohn et al., 2009; Speranza et al., 2020), including anti-viral (Cheng et al., 2005; Hsuan et al., 2009; Yang et al., 2012, Liu et al., 2015, 2016; Guo et al., 2020), anti-bacterial (Wu et al., 2019), anti-endotoxic (Wu et al., 2011; Zhang et al., 2019), anti-tumor (Hsuan et al., 2009), anti-inflammatory (Toshio et al., 2000; Ho and Chang, 2002; Chen et al., 2005), and immuno-regulatory properties (Toshio et al., 2000; Kizil et al., 2009). Băn Lán Gēn is the key ingredient of the traditional Chinese medicines “Isatis-Root Granule” and “Lianhua Qingwen Capsule,” which are used for treating SARS and SARS-CoV-2 infections (Li Z. et al., 2015; Li et al., 2020; Wu et al., 2020). The anti-viral properties of Băn Lán Gēn extracts may be attributed to the lignans and their glycosides (Li J. et al., 2015; Meng et al., 2017; Nguyen et al., 2017; Zhou et al., 2017; Xi et al., 2019; Cui et al., 2020). For instance, pinoresinol showed increased inhibitory effects on influenza viruses and Coxsackie virus B3 compared with other lignans *in vitro* (NorAzman et al., 2018). A pinoresinol monoglucoside, pinoresinol-*O*-β-D-glucoside, specifically inactivated multiple cellular signaling pathways elicited by viral infection and markedly reduced pro-inflammatory mediators production at the mRNA level (Li et al., 2018). Lariciresinol-4-*O*-β-D-glucoside significantly inhibited influenza A virus with suppress NF-κB activation and the expression of pro-inflammatory molecules, suggesting that lariciresinol-4-*O*-β-D-glucoside exerts anti-viral effects against influenza viruses (Li J. et al., 2015; Zhou et al., 2017). Clemastanin B showed notable inhibitory effects on influenza viruses (Yang et al., 2013). This suggests that lignans, especially their glycosides, are potential drug candidates for the development of anti-viral compounds. However, their use could be limited by the available number of cultivated plants, low-efficiency treatment procedures, and low purity levels (He et al., 2003; Tan et al., 2017; Li et al., 2019). Lignans are biosynthesized *via* the shikimate and phenylpropanoid pathways, which share a common precursor coniferyl alcohol with lignins in plants (Tashackori et al., 2019). In *I. indigotica*, coniferyl alcohol can be stereo-specific dimerized by a dirigent protein (DIR) to form pinoresinol and subsequently interacts with pinoresinol/lariciresinol reductases (PLR) and glycosyltransferases (UGT) to produce lariciresinol mono- or di-glycosides. Thus far, genes encoding phenylalanine ammonia-lyase (*PAL*), cinnamate-4-hydroxylase (*C4H*), 4-coumaroyl CoA-ligase (*4CL*), hydroxycinnamoyl-CoA shikimate hydroxycinnamoyl transferase (*HCT*), *PLR* involved in lignans biosynthesis have been identified in *I. indigotica* (Hu et al., 2011; Dong et al., 2015; Xiao et al., 2015). Of these, 19 *DIR* genes were annotated based on *I. indigotica* transcriptome, while additional functional analysis is needed to identify the key *DIR* genes involved in lignans (Li et al., 2014). In addition, Chen et al. (2021) isolated IiUGT71Bs, IiUGT71B5a/IiUGT71B5b, from *I. indigotica*, and analysis of enzymatic activities *in vitro* revealed IiUGT71Bs preferred catalyzing the glycosylation of pinoresinol. However, few functional studies further demonstrated their roles *in planta*, and what’s more, it remained unclear which IiUGT or IiUGTs involved in anti-viral lignan glycosides such as lariciresinol-4-*O*-β-D-glucoside and clemastanin B in *I. indigotica*. Thus, the UDP-glycosyltransferases involved in lignan glycosylation of *I. indigotica* remain to be elucidated.

UDP-glucose-dependent glycosyltransferases (UGTs) are a superfamily of enzymes that transfer a glycosyl moiety from UDP sugars to acceptor molecules, which play key roles in the stability, water-solubility, detoxification, and transportation of the plant’s secondary metabolites (Bowles et al., 2006). Plant *UGTs* contain a conserved protein domain comprising 44 amino acid residues at the C-terminal, which is known as the plant secondary product glycosyltransferase (PSPG) box. The homology of the amino acid sequence indicates that *UGTs* clustered in phylogenetic groups are more highly conserved in plants, while many of these clusters share similar sugar acceptors with other *UGTs*. For instance, the monolignol-specific *UGTs* families *UGT71*, *UGT72*, and *UGT88* are in the same clade (Gachon et al., 2005; Yonekura-Sakakibara and Hanada, 2011). However, only a few lignan-related *UGTs* have been characterized to date. UGT71A18 from *Forsythia* utilizes the furofuran-class lignans as substrates, especially (+)-pinoresinol and structurally related lignans *in vivo* (Eiichiro et al., 2010). In *Sesamum indicum*, UGT71A9 glycosylates the 2-hydroxyl of (+)-sesaminol, UGT94D1 glycosylates the 6′-hydroxyl of (+)-sesaminol 2-*O*-β-D-glucopyranoside, and UGT94G1 glycosylates the β*1*→2-hydroxyl of secoisolariciresinol monoglucoside (SMG) and (+)-sesaminol 2-*O*-β-D-glucosyl-(1→6)-*O*-β-D-glucoside (Noguchi et al., 2008; Ono et al., 2019). UGT74S1 from *Linum usitatissimum* L. can catalyze secoisolariciresinol with UDP-glucose to yield SMG and diglucoside (SDG) (Ghose et al., 2014). UGT71C1 uses pinoresinol and lariciresinol as substrates only forming monoglucosides in *Arabidopsis thaliana* (Atsushi et al., 2014). UGT71B5s from *I. indigotica* prefer glycosylating pinoresinol to form pinoresinol monoglycoside or diglycoside *in vitro* (Chen et al., 2021). However, these UGTs have a relatively low substrate specificity and poor catalytic efficiency for lariciresinol glycosylation, limiting their ability to synthesize lariciresinol glycosides. Therefore, it is important to identify new lignan-related UGTs from *I. indigotica.* In this study, we report three enzymes from *I. indigotica* for catalyzing the formation of lariciresinol glycosides *in vitro*, and demonstrated their roles in lignan biosynthesis using transgenic hairy roots. Our results indicate that *IiUGT4* is a key glycosyltransferase in clemastanin B biosynthesis in *I. indigotica*.

## Materials and Methods

### Plant Materials and Generation of *I. indigotica* Hairy Roots

The *I. indigotica* plant was grown in the suburbs of Beijing, China. The roots, stems, and leaves of *I. indigotica* were harvested in autumn, and stored at −80°C until used. The sterilized seeds of *I. indigotica* were grown on a Murashige and Skoog (MS) basal medium at 25°C for 25 days with a 12 h/12 h light/dark cycle. The leaves of sterile seeding were scratched with a knife, immersed in the modified *Agrobacterium rhizogenes* C58C1 suspension at 28°C for 10 min. The leaves were cocultured on MS basal medium at 25°C for 2 days, then transferred into an MS basal medium containing cefotaxime (400 mg⋅L^–1^), and the first batch hairy roots developed on the cut ends after 2 weeks of cocultivation. For the selection of transformants, PCR analysis were performed to verify the genes *rolb*, *rolc*, and *hpt* of the hairy roots with the genomic DNA as the template. Positive hairy roots were cultured on an MS liquid medium for 2 weeks, and then transferred to a 6,7-V liquid medium and cultured at 25°C in the dark.

### RNA Extraction, Transcriptome Sequencing, cDNA Cloning

Total RNA extraction, quantification, transcriptome sequencing and analysis were carried out as described by Zhao et al. (2016). The data has been deposited in the National Genomics Data Center (NGDC) (Accession Number: CRA004762). After annotations, 10 *IiUGTs* belonging to the *UGT71*, *UGT72*, and *UGT88* families whose members could catalyze lignan glycosylation were obtained. The coding sequences (CDS) of candidate genes were amplified from the cDNAs of hairy roots of *I. indigotica via* PCR with the specific primers and subsequently inserted into the pET-28a-HIS-MBP expression vector (Supplementary Table 5). Once they were positively sequenced, the constructs were transformed into *Escherichia coli* Rosetta cells.

### Heterologous Protein Expression and Activity Assay

The transformed cells described above were grown on the Luria-Bertani (LB) medium containing kanamycin (50 μg⋅mL^–1^) at 37°C. The cells were incubated with 0.5 mM Isopropyl thiogalactoside at 16°C for 16 h after OD_600_ reached 0.6. The cells were collected by centrifugation (5,000 × *g*, 10 min, 4°C), then resuspended in the lysis buffer (50 mM Tris-Cl, pH 7.4, 1 mM EDTA, 1 mM PMSF, 10% Glycerol), and sonicated for 5 s at 5 s intervals for 5 min. The lysates were centrifuged (12,000 × *g*, 15 min, 4°C), and the supernatant was collected as a crude enzyme. SDS-PAGE analysis was performed, and the proteins were visualized with Coomassie Brilliant Blue. The activity of IiUGT was assayed using lariciresinol as the substrate. The reaction mixture consisted of 300 μL crude protein, 1 μL 40 mM lariciresinol, and 2 μL 40 mM UDP-glucose. The reaction was incubated at 30°C for 12 h and stopped with 600 μL methanol. All reactions were centrifuged at RT (12,000 × *g*, 15 min), and analyzed by UPLC/Q-TOF-MS.

### NMR Analysis

To separate and identify the structure of the intermediate products, the reaction system was amplified in 30 mL of crude IiUGT1 protein, 400 μM UDP-glucose, and 200 μM lariciresinol. The reaction mixture was extracted with two volumes of methanol, and dissolved with 2 mL methanol after pressurizing and drying the organic phase. Acetonitrile and water (1:3, V/V) was used as a mobile phase to separate the reaction products in a SHIMADZU UFPLC system at a flow rate of 10 mL/min with a 210 nm UV wavelength, using a C18 20 mm × 250 mm column. The separated products were determined using UPLC/Q-TOF-MS, ^1^H NMR, and ^13^C NMR. The NMR spectrum data of the former were consistent with the reported NMR spectrum (+)-lariciresinol-4-*O*-β-D-glucoside; The NMR spectrum data of the latter were consistent with the reported NMR spectrum (+)-lariciresinol-4′-*O*-β-D-glucoside (Masataka and Masao, 1993; Li J. et al., 2015; Supplementary Table 4).

### Sequence Alignment and Phylogenetic Analysis

The gene sequences coding region and resulting amino acid sequences were analyzed with Vector NTI Advance 11.5.3. The position of the plant UGT conserved motifs was determined using the position scanning tool^1^ of the ExPASy network interface. The reference sequences were downloaded from the GenBank database and the protein sequences were aligned using the MAFFT program. A neighbor-joining phylogenetic tree for IiUGTs and other plant UGTs was constructed using the *p*-distance algorithm in MEGA 7.0 with 1000 bootstrap replicates.

### Purification and Catalytic Parameters of Recombinant IiUGT Proteins

The recombinant proteins were purified using Ni-NTA resin and concentrated using Amicon-Ultra-0.5 Ultracel-10 k (BRAND Millipore). The purity of the recombinant IiUGTs was assessed with 10% SDS-PAGE, and the concentration was determined with a Bradford Protein Assay kit. During the initial screening, each reaction contained 50 mM Tris-Cl buffer, 200 μM substrate, 400 μM UDP-glucose, and 20 μg purified protein (100 μL reaction volume). The reaction was then incubated at 35°C for 60 min and stopped with 200 μL Methanol. The optimum reaction temperature was between 25 and 55°C, and the optimum pH value was between 4.0 and 10.8. Citric acid-sodium citrate, sodium phosphate, Tris-Cl, and sodium carbonate were used for pH ranges of 4.0–6.0, 6.0–8.0, 7.0–9.0, and 9.0–10.8, respectively. For kinetic studies, the recombinant IiUGTs were incubated in the volume of 100 μL with 1.6 mM UDP-glucose. A minimum of eight substrate concentrations between 20 and 800 μM were used. The kinetics data were fitted to the Michaelis-Menten equation with GraphPad Prism 6.0 software to obtain the kinetic parameters of the enzyme reaction.

### Expression Analysis by qRT-PCR

Total RNA was extracted from different *I. indigotica* tissues using TRIzol reagent (Invitrogen), while the first-strand cDNA was synthesized with total RNA (200 ng each). qRT-PCR analysis was performed using SYBR Premix Ex Taq II and quantified on Roche LightCycler 480 to determine the transcript abundance of the candidate *IiUGTs*. The primers are listed in Supplementary Table 5, while the melting curves of each primer pair were a single peak. Relative expression levels were determined using the 2^–ΔΔ*Ct*^ method.

### Subcellar Localization of IiUGTs

Coding regions without IiUGT stop codons were amplified and inserted into the corresponding sites of a modified pCAMBIA1300, fusing them with the green fluorescent protein (cGFP) coding sequence. Recombinant vectors carrying *IiUGT* and the empty vector pCAMBIA1300-cGFP were transferred into the *Agrobacterium tumefaciens* strain GV3101. Combinations of *A. tumefaciens* strains were combined with 6-week-old *Nicotiana benthamiana* leaves after 2 days, the green fluorescent protein (GFP) fluorescence of *N. benthamiana* leaves was captured using a Zeiss LSM 510 META confocal microscope.

### Generating RNAi and Overexpression Transgenic Hairy Root Lines

The non-conserved regions of *IiUGT1*, *IiUGT4*, and *IiUGT71B5a* were amplified from the cDNA of wild-type hairy roots to design a double-stranded hairpin RNA. The primers were listed in Supplementary Table 5. The fragments were then cloned into vector pZH02 (pCAMBIA1300-Based). To assess the overexpression of candidate *IiUGTs*, the full-length open reading frame (ORF) of *IiUGT1*, *IiUGT4*, *IiUGT71B5a* were amplified from the cDNAs of hairy roots and cloned into the vector pCAMBIA1300-Super. After validating the sequences, the constructs were used to transform the *A. rhizogenes* strain C58C1. Positive transformants were cultured on the LB medium, and identified *via* PCR analysis. The *I. indigotica* hairy roots were generated using *A. rhizogenes* according to the same processes previously described. The leaf explants were co-cultivated for 2 days with the engineered strains on an MS basal medium and subsequently transferred into an induction media containing cefotaxime (400 mg⋅L^–1^) and hygromycin (50 mg⋅L^–1^) after they were washed. After 2 weeks of cocultivation, hairy roots were observed on the cut ends of the leaves, and successful transformants were screened for positive lines *via* PCR analysis. The positive hairy roots were removed from the explants, cultured on the MS medium, and maintained as independent lines. To facilitate hairy root growth, we did not add hygromycin and continuously reduced the concentration of cefotaxime.

### Verification of IiUGT-Overexpressing Hairy Roots *via* Western-Blot Analysis

After 0.05 g of fresh hairy roots were ground into liquid nitrogen, the mixture was boiled for 10 min using 100 μL 2.5 × SDS Buffer (50 mM Tris-Cl, pH 6.8, 0.4% Bromophenol blue, 8% SDS, 40% glycerol) to extract the total proteins. Aliquots of 8 μL total protein per line were separated on a 10% SDS-PAGE gel and blotted to a PVDF membrane. After blotting, the PVDF membrane was washed three times with a PBS buffer, enclosed with a 50 mL PBS buffer, and shaken at RT for 60 min. The blots were probed with Anti-DYKDDDDK Mouse Monoclonal Antibodies (1:2000, TransGen Biotech, Beijing, China) in a 2.5 mL PBS buffer containing skimmed milk powder at 4°C for 1 h, washed with PBS buffer three times, reacted with Goat Anti Mouse lgG HRP (1:5000, TransGen Biotech, Beijing, China) at 4°C for 45 min, and finally exposed to X-ray film using ECL Detection Reagent.

### Lignan Glycosides Content Analysis Using UPLC-MS/MS

Lignan glysoside content was analyzed on UPLC-MS/MS system. The hairy roots used in the lignan glycoside content analysis were harvested from a 6,7-V liquid medium after 30 days. After freeze-drying the extract and grinding it into a powder, we extracted 50 mg powdered samples using 1.5 mL methanol under sonication for 45 min and centrifuged (12,000 × *g*, 15 min). The supernatant was carried out on a C18 column (2.1 mm × 10 mm, 1.8 μm, Waters) with 0.1% formic acid-acetonitrile (A) and 0.1% formic acid-water (B) as mobile phases and run at 0.6 mL min^–1^ according to the following specifications: 0 min, 5% A; 1 min, 25% A; 3.5 min, 40% A; 4 min 5% A; 4∼6 min, 5% A. Multiple monitoring methods were used to assess quantification, while the selected *m/z* transitions were 179.0→146.2 at 1.99 min for coniferyl alcohol, 357.0→150.8 at 3.32 min for pinoresinol, 361.1→164.9 at 2.71 min for secolariciresinol, 341.2→179.0 at 1.29 min for coniferin, 359.1→328.0 at 2.84 min for lariciresinol, 357.0→122.0 at 3.58 min for matairesinol, 680.9→519.0 at 1.79 min for pinoresinol diglucoside, 521.2→329.0 at 2.02 min for lariciresinol-4-*O*-β-D-glucoside, 521.2→329.0 at 2.17 min for lariciresinol-4′-*O*-β-D-glucoside, and 683.2→521.2 at 1.58 min for clemastanin B (Supplementary Table 6).

### Homology Modeling and Molecular Docking

The 3D structure of IiUGT4 were modeled using SWISS-MODLE^2^ based on the UGT71G1 crystal structure (PDB ID: 2acv), the sequence of which was 40.8% similar to IiUGT4 and had the highest GMQE (Global Model Quality Estimation) value. The molecular docking between IiUGT4 and lariciresinol was assessed using SYBYL X-1.2 (Li et al., 2018), and the residues H15, D124, F151, A207, S289, H373, W376, S378, Y395, and E397 were selected based on these results.

### Site-Directed Mutagenesis of IiUGT

The site-directed mutagenesis of IiUGT4 at the residues was cloned *via* PCR using pET28a-HIS-MBP-IiUGT4 as the template. The primers are listed in Supplementary Table 5. The PCR products were digested with a DMT enzyme and transformed into DMT *E. coli*. The mutants were confirmed *via* sequencing and transformed into Rosetta (DE3) *E. coli* for expression.

## Results

### Screening of Candidate liUGTs and Sequence Analysis for Generating Clemastanin B in *I. indigotica*

**FIGURE 1:**
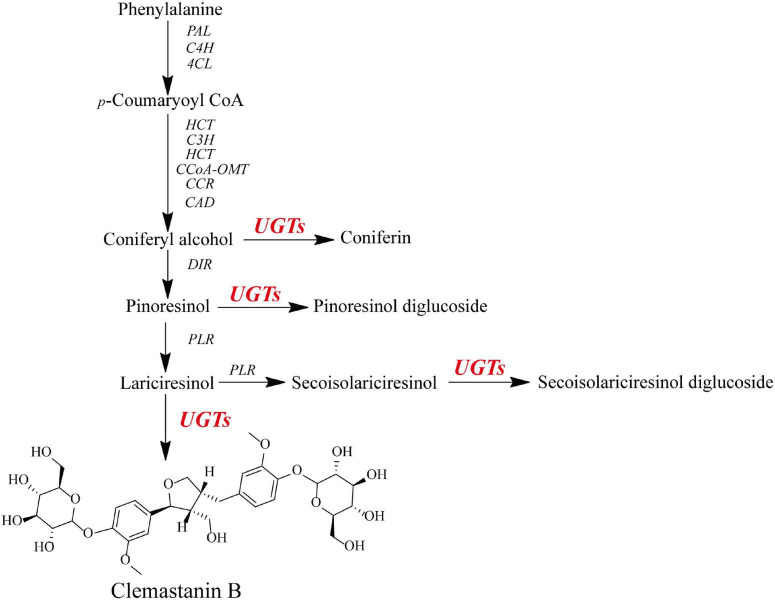
Schematic for the putative biosynthetic pathway of *O*-glycosylation toward lignans in *I. indigotica*.

To identify the genes caplable for the *O*-glycosylation of lignans, a normalized cDNA library were constructed using a total RNA sample taken from the roots, stems, leaves, and cultured hairy roots of *I. indigotica* in equal proportions. This generated 57,072 unigenes with an average length of 827.69 bp after assembly (Supplementary Table 1), which were all annotated according to the public database (Figure 1). After annotation and homologous searching, fourteen *IiUGTs* belonging to the *UGT71*, *UGT72*, and *UGT88* families were obtained as putative glycosyltransferases. Given that clemastanin B also accumulates in the hairy roots of *I. indigotica*, the hairy root cDNA was as a template to clone candidate *IiUGT* genes. Finally, the ORFs of ten candidate *IiUGTs* were amplified for further investigation (Supplementary Figure 1 and Supplementary Table 2).

**FIGURE 2:**
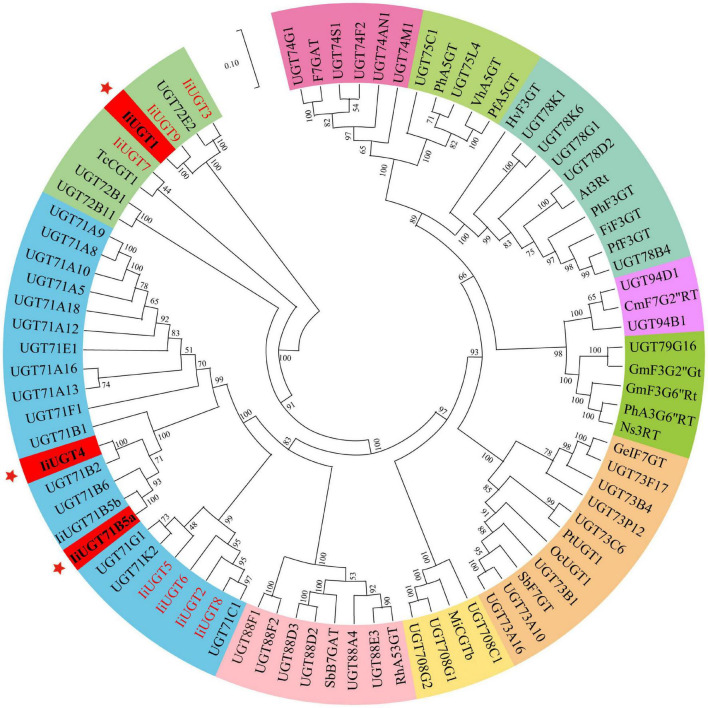
Phylogenetic relationships between candidate *IiUGTs* and their close relatives. *IiUGT* genes from this study were in red or blod in black. A Neighbor-Joining phylogenetic tree was constructed the tree with 1000 bootstrap replicates by MEGA7. The GenBank accession numbers of UGT proteins in the tree are in Supplementary Table 2. The key genes whose functions were characterized in this study were marked with *.

A phylogenetic tree was constructed to examine how the ten IiUGTs were related to previously characterized UGTs under their protein sequences (Supplementary Table 3). IiUGT2, IiUGT5, IiUGT6, and IiUGT8 were grouped into a clade with UGT71C1 (Figure 2). IiUGT4 and IiUGT71B5a belonged to the UGT71B family, which formed a clade with plant UGTs from different species that utilize phenylpropanoids as substrates. Additionally, the amino acid sequences of IiUGT4 were 81.31% similar to the phenylpropanoid glycosylation UGT71B2 gene of *A. thaliana* (NP_188813.1). Similarly, IiUGT71B5a was 65.70% similar to the phenylpropanoid glycosylation UGT71B6 gene of *A. thaliana* (NP_188815.2). However, IiUGT1, IiUGT3, IiUGT7, and IiUGT9 were categorized under the UGT72 family, which could catalyze the glycosylation of phenylpropanoids. IiUGT1, IiUGT3, and IiUGT9 were grouped into a clade with the mono-lignol UGT72E from *A. thaliana*, while IiUGT7 was more closely related to TcCGT1 from *Trollius chinensis*. The results suggest that these candidate IiUGTs may play specific roles during the glycosylation of lignans or other phenylpropanoids in *I. indigotica*.

### Enzymatic Activities of Candidate IiUGTs

**FIGURE 3:**
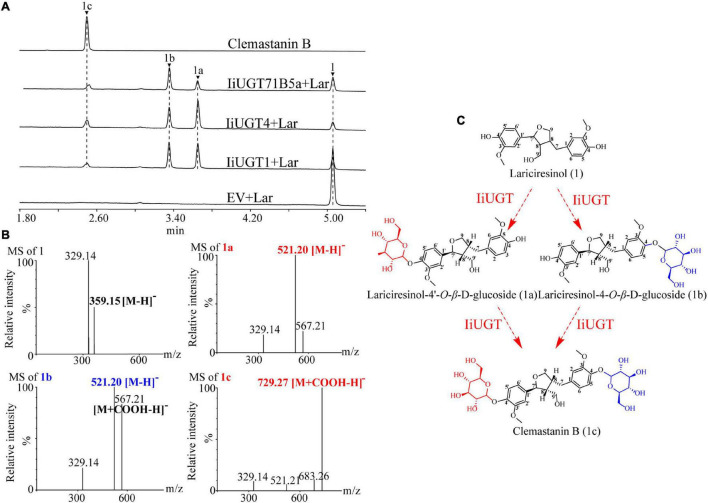
The hypothetical biosynthetic pathway for clemastanin B in *I. indigotica* and functional characterization of candidate IiUGT proteins. **(A)** UPLC/Q-TOF-MS analysis of candidate IiUGT enzymatic reaction products against lariciresinol. The enzyme reactions of crude proteins of *E. coil* carrying empty vector (EV), IiUGT1, IiUGT4, IiUGT71B5a were assayed with UDP-glucose as the sugar donor and Lar (lariciresinol) as the sugar acceptor. (1) lariciresinol; (1a) lariciresinol-4′-*O*-β-D-glucoside; (1b) lariciresinol-4-*O*-β-D-glucoside; (1c) clemastanin B. **(B)** MS spectra of the products in negative mode. **(C)** A proposed biosynthetic pathway for clemastanin B. The monoglycoside products were purified in this study and identified by NMR (Supplementary Table 4).

The 10 *IiUGT* genes were subsequently cloned into a prokaryotic expression vector for further functional analysis. SDS-PAGE analysis confirmed that the IiUGTs recombinant protein were successfully expressed with their expected MW, following induction with IPTG (Supplementary Figure 2). The recombinant IiUGT enzymes were assayed with lariciresinol and UDP-glucose as substrates to investigate the functional proteins displaying *O*-glucosylation activity against lariciresinol from the candidate *IiUGTs* (Figure 3 and Supplementary Figure 3). Three of the recombinant enzymes reacted with lariciresinol when the sugar donor UDP-glucose was present, resulting in a new peak identical to the authentic clemastanin B identified by the UPLC/Q-TOF-MS analysis (Figure 3B) (Yang et al., 2013). The results demonstrated that these three enzymes catalyzed glycosylation at the 4-hydroxy group and the 4′-hydroxy group of lariciresinol into lariciresinol diglucoside (clemastanin B). The UPLC/Q-TOF-MS analysis also found two new peaks exhibiting the [M + COOH-H]^–^ ions at *m/z* 567.21 and the [M-H]^–^ ions at *m/z* 521.20, both of which had longer retention times than clemastanin B in these three reactions. The products were separated and analyzed by nuclear magnetic resonance (NMR) to clarify the structure of the intermediate lariciresinol products. NMR analysis revealed that the former was consistent with (+)-lariciresinol-4-*O*-β-D-glucoside, while the other was consistent with (+)-lariciresinol-4′-*O*-β-D-glucoside (Supplementary Table 4). These results demonstrated that these three enzymes could efficiently glycosylate the 4-hydroxyl and 4′-hydroxyl of lariciresinol into lariciresinol monoglucosides [((+)-lariciresinol-4-*O*-β-D-glucoside, (+)-lariciresinol-4′-*O*-β-D-glucoside)] and diglucoside (clemastanin B) (Figure 3C).

The enzymes were also tested with other potential native substrates, including coniferyl alcohol, pinoresinol, secoisolariciresinol, and matairesinol with UDP-glucose as a sugar donor. Although the three enzymes exhibited a considerable affinity for the glycosylation of native substrates, there were some differences in their catalytic products. The three enzymes converted coniferyl alcohol to form coniferin compared with the retention time of the control, and exhibited capable catalytic activity to produce mono-glucosides of secoisolariciresinol and matairesinol (Supplementary Figure 4). IiUGT1 and IiUGT71B5a were able to glycosylate pinoresinol to form pinoresinol monoglucoside and diglucoside, however, IiUGT4 was more likely to form pinoresinol monoglucoside (Supplementary Figure 4).

### Biochemical Characterization of Recombinant liUGTs Expressed in *Escherichia coli*

Purified recombinant proteins were analyzed to assess their substrate specificity and catalytic properties, which helped validate the enzymatic properties of IiUGT proteins (Supplementary Figure 5). The optimum reaction temperatures of IiUGTs were determined using lariciresinol and UDP-glucose as the substrates, with all enzymes experiencing maximum activity at 35°C (Supplementary Figure 6). While the amino acid sequences of IiUGT4 and IiUGT71B5a are 60.90% similar, their pH values differed. The optimal pH for IiUGT4 activity was 9.0 and had low activity between pH 4.0 and 6.0, while the optimal pH value for IiUGT71B5a was 8.0 at the Na_2_HPO_4_-NaH_2_PO_4_ buffer. IiUGT1 exhibited at least 80% of its maximum activity at pH values of 7.0 and 9.2 at Na_2_HPO_4_-NaH_2_PO_4_ and Tris-Cl buffer (Supplementary Figures 6A–C).

The kinetic properties of IiUGTs were determined by analyzing the linear range of the enzymatic reaction. The apparent *K*_*m*_ value of IiUGT4 for lariciresinol (80.92 μM) was lowest: 12.50 times and 2.10 times greater than the affinity of IiUGT1 and IiUGT71B5a, respectively (Table 1). The *k_*cat*_/K_*m*_* value of IiUGT4 for lariciresinol (785.91 M^–1^s^–1^) was nearly double that of IiUGT71B5a (346.78 M^–1^s^–1^), but was similar to that of IiUGT1 (748.75 M^–1^s^–1^). The *K*_*m*_ value of IiUGT4 for pinoresinol was about 5 times and 2 times lower than that of IiUGT1 and IiUGT71B5a, respectively, but the *k_*cat*_/K_*m*_* value of IiUGT4 for pinoresinol did not significantly differ from the value obtained using IiUGT71B5a, and was similar to that of IiUGT1. Although the apparent *K*_*m*_ value of IiUGT4 for matairesinol was the lowest, there was no significant difference in activities between IiUGT1 and IiUGT4 with regard to *k_*cat*_/K_*m*_* values. The *K*_*m*_ values of IiUGTs for secoisolariciresinol were similar, but the *k_*cat*_/K_*m*_* values of IiUGT1 and IiUGT4 for pinoresinol were both 1.50 times higher than that of IiUGT71B5a. These results suggest that the three IiUGTs may play multiple roles in catalyzing the glycosylation of different substrates in *I. indigotica.* The apparent *K*_*m*_ value of IiUGT4 for lariciresinol (80.92 μM) was 2.2 times higher than that of matairesinol (36.44 μM). However, the *k_*cat*_/K_*m*_* values (785.91 M^–1^s^–1^ for lariciresinol, 740.36 M^–1^s^–1^ for matairesinol) did not differ significantly between lariciresinol and matairesinol. Given that matairesinol glycosides were barely detectable in *I. indigotica*, IiUGT4 may be the more efficient enzyme compared with IiUGT1 and IiUGT71B5a for the glycosylation of lariciresinol *in vivo*.

**TABLE 1:** Kinetic parameters of candidate recombinant IiUGTs.

Substrates	Km (uM)	Vmax (nmol/min.mg)	Kcat (s^–1^)	Kcat/Km (M^–1^s^–1^)
**IiUGT1**				
Lariciresinol	1012 ± 157.7	146.10 ± 45.3	0.75	748.75
Pinoresinol	626 ± 55.23	168.00 ± 11.23	0.40	647.38
Matairesinol	190.20 ± 43.28	25.47 ± 11.98	0.15	798.80
Secoisolariciresinol	80.97 ± 13.37	41.97 ± 2.94	0.07	883.42
Coniferyl alcohol	188.9 ± 28.8	8.119 ± 0.493	0.008	46.86
**IiUGT4**				
Lariciresinol	80.92 ± 25.33	38.07 ± 3.83	0.06	785.91
Pinoresinol	128.3 ± 20.97	34.09 ± 2.20	0.06	443.86
Matairesinol	36.44 ± 6.00	16.15 ± 0.76	0.03	740.36
Secoisolariciresinol	94.91 ± 21.4	43.93 ± 4.39	0.07	773.21
Coniferyl alcohol	141.4 ± 18.46	7.602 ± 0.354	0.008	58.81
**IiUGT71B5a**				
Lariciresinol	170.00 ± 28.89	35.29 ± 2.37	0.06	346.78
Pinoresinol	229.30 ± 33.79	64.25 ± 4.20	0.11	468.08
Matairesinol	54.68 ± 8.71	17.60 ± 1.00	0.03	537.69
Secoisolariciresinol	101.10 ± 18.72	35.43 ± 2.98	0.06	585.42
Coniferyl alcohol	216.8 ± 20.21	8.80 ± 0.34	0.009	44.18

*The results are the means and ± SD of three independent experiments.*

### Subcellular Localization Analysis of IiUGTs

**FIGURE 4:**
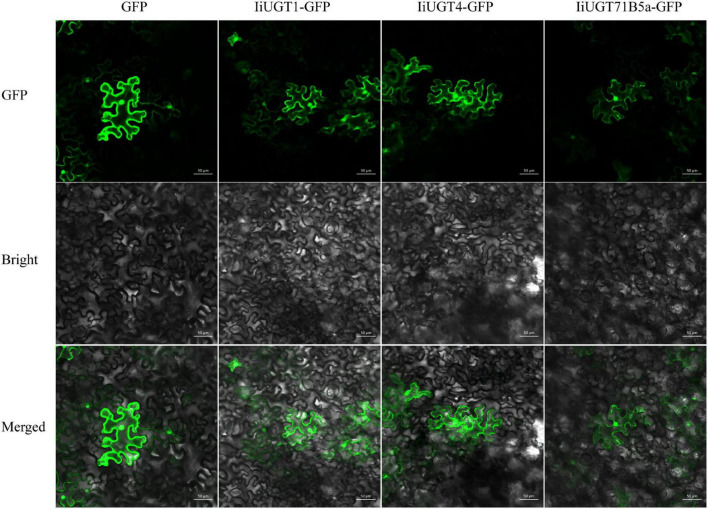
Subcellular localization of IiUGTs fused with GFP at the C-terminus in *N. benthamiana*. GFP, GFP channel; Bright, light microscopy image; Merged, merged image of the GFP and Bright channels. Scale bars are 50 μm.

We were unable to predict the localization of the IiUGT proteins because no signal peptide was found in the full amino acid sequence using the SignalP 3.0 Server. Therefore, the recombinant IiUGT fused with GFP at the C-terminus were transiently expressed in tobacco leaves to determine the subcellar localization of IiUGTs. The GFP-tagged IiUGT was distributed throughout the cytoplasm and the nucleus (Figure 4). These results were similar to other UGTs in plants, including AcUFGT3a, which regulates anthocyanin accumulation in red-fleshed kiwifruit, and OsIAGLU, which mediates crosstalk between auxin and abscisic acid to regulate seed vigor in *rice* (Liu et al., 2018; He et al., 2020).

### Expression Profiling of Candidate liUGT Genes in *I. indigotica*

**FIGURE 5:**
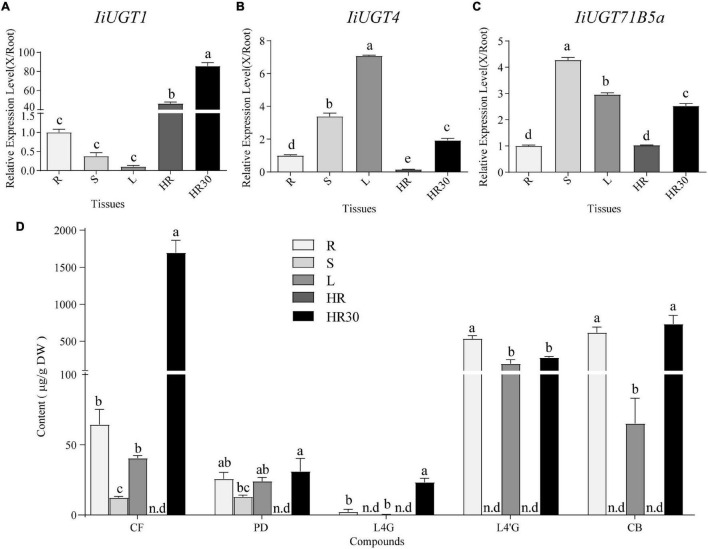
qRT-PCR analysis of *IiUGT1*, *IiUGT4*, *IiUGT71B5a* expression patterns in *I. indigotica* and the accumulation of lignan glycosides in *I. indigotica.* Relative expression levels of *IiUGT1* **(A)**, *IiUGT4* **(B)** and *IiUGT71B5a* **(C)** in *I. indigotica* were performed as described in the experimental procedures using total RNAs extracted from different organs. The tissue samples are listed as follows: R, roots; S, stems; L, leaves; HR, hairy roots cultivated in MS liquid medium for 2 weeks; HR30, hairy roots cultivated in 6,7-V liquid medium for 30 days. The expression of *IiUGT* in root tissue was defined as 1.0. **(D)** Quantities of lignan glycosides from different tissues of *I. indigotica*. The tissue samples are listed as follows as above. CF, coniferin; PD, pinoresinol diglucoside; L4G, lariciresinol-4-*O*-β-D-glucoside; L4′G, lariciresinol-4′-*O*-β-D-glucoside; CB, clemastanin B; n.d, not detected. All data represent the mean ± standard deviation (SD) of three biological replicates. Different letters above the error bars indicate significant differences (*p* < 0.05) according Tukey’s test.

To investigate the roles of the three *IiUGTs in planta*, qRT-PCR analysis were performed to analyze the expression profiles and determine expression levels in different tissues of *I. indigotica*, including roots, stems, leaves, and hairy roots. *IiUGT1* was primarily expressed in the hairy roots of *I. indigotica*, while its transcription level was almost undetectable in stems and leaves (Figure 5A). Differing from *IiUGT1*, *IiUGT4*, and *IiUGT71B5a* had similar expression patterns, with higher expression levels in the above-ground parts than in the roots, but lower in MS medium-cultured hairy roots (Figures 5B,C). Interestingly, transcription levels of *IiUGT* genes were up-regulated in 6,7-V medium-cultured hairy roots for 30 days. These results indicate that *IiUGT1* may contribute primarily to specialized metabolism in hairy roots, while the other two *IiUGTs* play multiple roles in hairy roots and other tissues.

To explore the roles of these *IiUGT* genes in the biosynthesis of lignan glucosides, we examined the amount of five common lignan glycosides in different organs and hairy roots, and found a larger accumulation of coniferin (CF), pinoresinol diglucoside (PD), lariciresinol-4-*O*-β-D-glucoside (L4G), lariciresinol-4′-*O*-β-D-glucoside (L4′G), and clemastanin B (CB) in both roots and leaves compared with in stems (Figure 5D). This finding indicates that accumulation of lignan glycosides occurred in both roots and leaves. Specifically, lariciresinol-derivatived glycosides, especially lariciresinol-4′-*O*-β-D-glucoside and clemastanin B, primarily accumulated in roots and leaves. This was consistent with the higher observed transcription levels of *IiUGT4* and *IiUGT71B5a* in the corresponding tissues, while a comparable amount of coniferin and pinoresinol diglucoside also accumulated in the stems, not only in the roots and leaves. Transcription profiles of these *IiUGTs* were not strictly consistent with the detected accumulation of lignan glycosides, though it is not rare that the activity of plant UGTs *in vivo* is inconsistent with its *in vitro* activity, which has been reported in plant UGT enzymes, such as AtUGT73C6, MtUGT78G1, and LjUGTs (Peel et al., 2009; Husar et al., 2011; Yin et al., 2017). These observations suggest that the heterogeneities and functional diversification of UGT families contribute to the complex glycosylation network of lignan glycosides *in planta*. Therefore, *in vivo* validation is the most effective method to uncover the role of UGTs *in planta*.

### Knock-Down of Candidate liUGTs Decreased Lignan Glycosides Content in Hairy Roots

**FIGURE 6:**
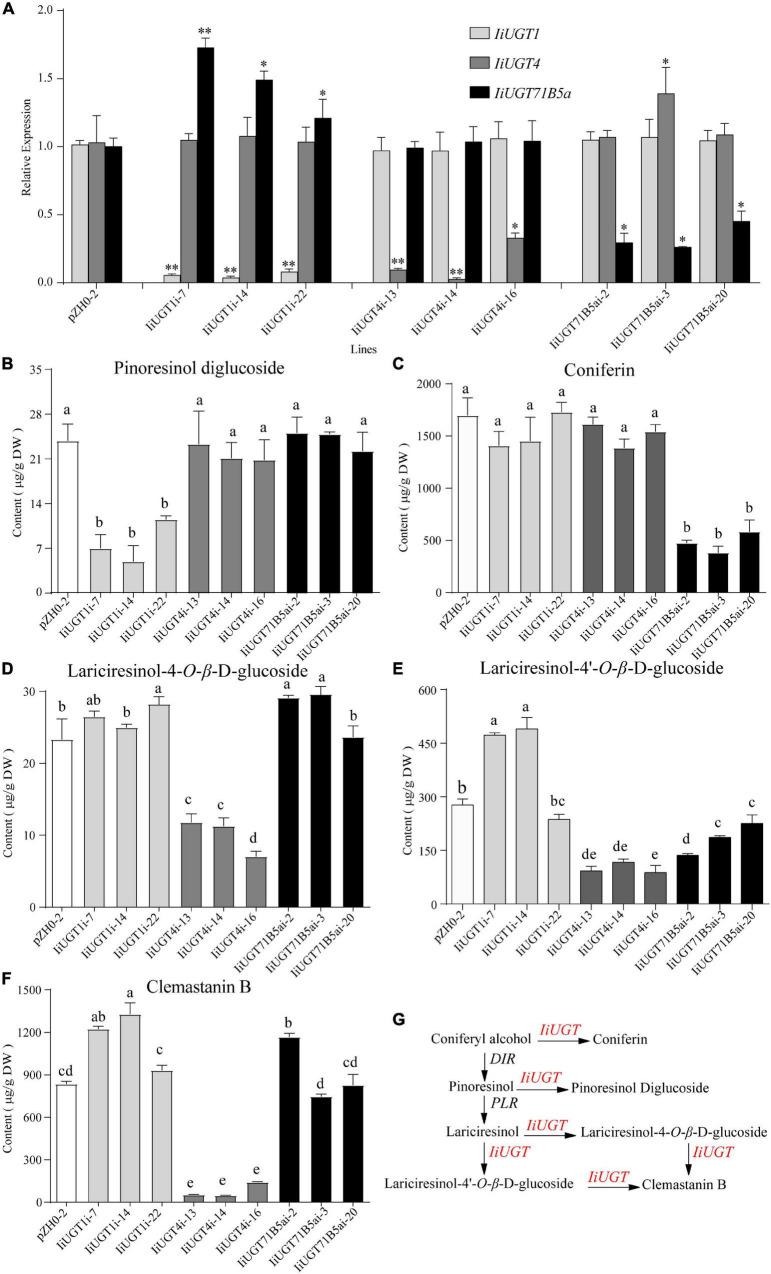
RNA interference silencing of *IiUGT1*, *IiUGT4* and *IiUGT71B5a* in *I. indigotica* hairy roots. **(A)** Relative mRNA levels of *IiUGT* in RNAi hairy roots was measured by monitoring relative transcript levels by qRT-PCR. Effect of *IiUGT* gene silencing on the levels of Pinoresinol diglucoside **(B)**, Coniferin **(C)**, Lariciresinol-4-*O*-β-D-glucoside **(D)**, Lariciresinol-4′-*O*-β-D-glucoside **(E)**, Clemastanin B **(F)**. Standard errors were calculated from three biological replicates. Different letters above the error bars indicate significant differences (*p* < 0.05). Statistical sinificance levels between the variable groups and the control group were calculated using one-way ANOVA test and Tukey’s multiple comparisons test (*p < 0.05 and ^**^p < 0.01). **(G)** Proposed pathway from coniferyl alcohol to lignan glycosides in *I. indigotica*.

Previous studies have demonstrated that the hairy roots of *I. indigotica* can accumulate a comparable amount of lignans (primarily in glycoside form), including mono- and di-glycosides of lariciresinol. This suggests that the hairy roots are the ideal biosystem to analyze the biosynthetic pathway of lignans and functionally characterize the candidate enzyme genes in *I. indigotica* (Chen et al., 2015; Zhang et al., 2016; Tan et al., 2017). To further insight into the role of the three *IiUGTs* in lignan glycosylation in *I. indigotica*, RNAi strategies were used to generate gene knock-down transgenic lines of *IiUGTs* (Supplementary Figure 7). Transgenic nature of hairy roots were proved using the PCR analysis for *rolb*, *rolc*, and *hpt* genes. The presence of double-stranded hairpin RNA construct in *IiUGT-RNAi* lines were confirmed by PCR with the specific primers (Supplementary Figure 8 and Supplementary Table 5). qRT-PCR analysis demonstrated that transcript levels of the target genes were significantly downregulated in three independent hairy root lines, but the transcript levels of *IiUGT71B5a* were up-regulated in the *IiUGT1-RNAi* lines (Figure 6A).

Analysis of the metabolite contents demonstrated that the downregulation of *IiUGT1* produced significantly lower levels of pinoresinol diglucoside compared with the control line. No obvious changes were observed when either *IiUGT4* or *IiUGT71B5a* was downregulated (Figure 6B). When the transcription levels of *IiUGT1* decreased, the accumulation of coniferin content was unaffected, however, the contents of lariciresinol-4-*O*-β-D-glucoside, lariciresinol-4′-*O*-β-D-glucoside, and clemastanin B in some *IiUGT1-RNAi* lines were significantly upregulated compared with the controls (Figures 6C–F). For instance, line *IiUGT1i-14* had the lowest *IiUGT1* mRNA level and accumulated the least pinoresinol diglucoside (4.92 μg⋅g^–1^DW). This was a 4.85-fold decrease from the control (23.82 μg⋅g^–1^DW), but its lariciresinol-4′-*O*-β-D-glucoside, and clemastanin B contents were approximately 1.3 times higher than the controls, and it did not exhibit a difference in coniferin content. This indicates that *IiUGT1* could be responsible for the glycosylation of pinoresinol in *I. indigotica*. Increases in the contents of lariciresinol-4-*O*-β-D-glucoside, lariciresinol-4′-*O*-β-D-glucoside, and clemastanin B in transgenic lines could be positively correlated with *IiUGT71B5a* expression levels (Figures 6A,D,E,F).

The knock-down of *IiUGT4* resulted in significantly lower lariciresinol-4-*O*-β-D-glucoside, lariciresinol-4′-*O*-β-D-glucoside, and clemastanin B contents, but did not affect pinoresinol diglucoside and coniferin contents compared with the control lines (Figures 6B–F). In *IiUGT4i*-14, lignan glycosides contents decreased to 48.28, 33.25, and 5.60% for lariciresinol-4-*O*-β-D-glucoside, lariciresinol-4′-*O*-β-D-glucoside, and clemastanin B, respectively, along with a 92% decrease in *IiUGT4* transcript compared with the control lines. Therefore, *IiUGT4* could be primarily involved in converting lariciresinol to clemastanin B in *I. indigotica*.

In *IiUGT71B5a* knock-down lines, coniferin content was significantly decreased, while the contents of other lignan glycosides, pinoresinol diglucoside, and clemastanin B, were not significantly decreased compared with the control lines (Figures 6B,C,F). Furthermore, coniferin content was not affected in either the *IiUGT1-RNAi* lines or the *IiUGT4-RNAi* lines. Therefore, *IiUGT71B5a* was primarily involved in the biosynthesis of coniferin in *I. indigotica*. Contents of lariciresinol-4′-*O*-β-D-glucoside significantly decreased in *IiUGT71B5a-RNAi* lines, suggesting that *IiUGT71B5a* could also contribute to the biosynthesis of lariciresinol-4′-*O*-β-D-glucoside.

The results demonstrated *IiUGT4* could be involved in the conversion of lariciresinol to relevant glycosides, while *IiUGT1* and *IiUGT71B5a* could be responsible for the glycosylation of pinoresinol and coniferyl alcohol in the hairy roots of *I. indigotica*, respectively (Figure 6G).

### Overexpression of Candidate IiUGTs Increased Lignan Glycosides Contents in Hairy Roots

These *IiUGTs* could be the principal enzyme involved in the glycosylation of lignans, making it important to assess the effects of their overexpression in *I. indigotica*. Therefore, the full-length ORF of *IiUGTs* were separately cloned into the pCAMBIA1300sp-cflag vector for the *Agrobacterium*-mediated transformation of *I. indigotica* leaves to generate transgenic hairy root lines (Supplementary Figure 9).

**FIGURE 7:**
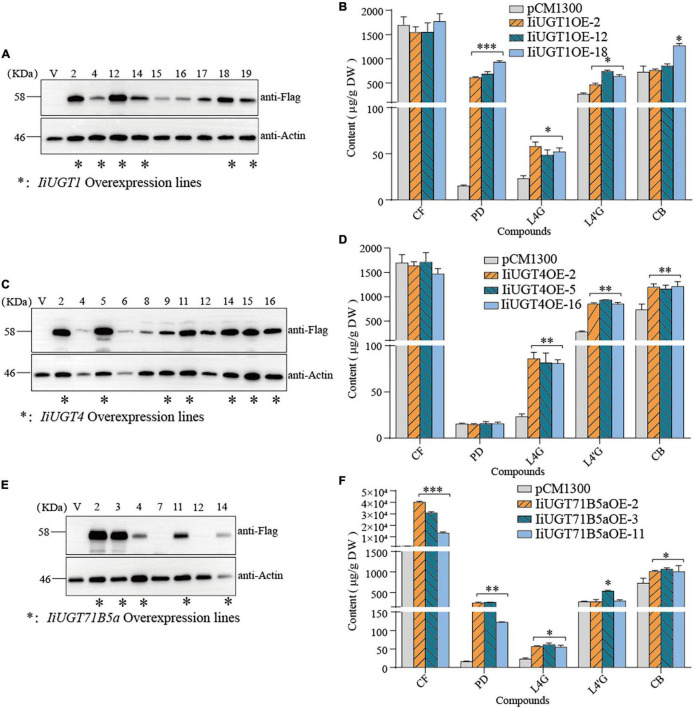
Overexpression of *IiUGTs* in *I. indigotica* hairy roots. **(A,C,E)** Western-blot analysis showing *IiUGT* transcript expression. Effect of *IiUGT1* **(B)**, *IiUGT4* **(D)** and *IiUGT71B5a* **(F)** overexpressing on the contents of coniferin (CF), pinoresinol diglucoside (PD), lariciresinol-4-*O*-β-D-glucoside (L4G), lariciresinol-4′-*O*-β-D-glucoside (L4′G), clemastanin B (CB). Standard errors were calculated from three biological replicates. Statistical sinificance levels between the variable groups and the control group were calculated using one-way ANOVA test and Tukey’s multiple comparisons test (*p < 0.05, ^**^p < 0.01, and ^***^p < 0.001).

PCR analysis showed that transgenic hairy roots lines contatin *rolb*, *rloc* and *hpt* genes, and the exogenous *IiUGT* gene was successfully integrated into *IiUGT*-OE lines (Supplementary Figure 10). To further assess the accumulation of the Flag-tagged *IiUGT* in *IiUGT*-OE lines, a western blot analysis were performed using an anti-flag antibody and detected three strong cross-reaction signals corresponding to the expected sizes of the *IiUGT*-flag fusion protein in some of the transgenic lines. These positive transgenic lines were used for further analysis (Figures 7A,C,E).

Metabolite analysis showed that *IiUGT1*-OE lines had significantly higher levels of pinoresinol diglucoside. Compared with the control line which accumulated pinoresinol diglucoside at 15.58 μg⋅g^–1^ dry weight (DW), the *IiUGT1*-OE lines accumulated up to 39.72 and 60.09 times as much pinoresinol diglucoside than the control. In the *IiUGT1*-OE lines, the lariciresinol-4-*O*-β-D-glucoside, lariciresinol-4′-*O*-β-D-glucoside contents also increased, while we observed no obvious changes in coniferin content in these lines (Figure 7B).

*IiUGT4-*OE lines differed from *IiUGT1*-OE lines in that the content of lariciresinol-4-*O*-β-D-glucoside, lariciresinol-4′-*O*-β-D-glucoside, and clemastanin B significantly increased but did not affect coniferin and pinoresinol diglucoside accumulation (Figure 7D). *IiUGT4OE*-5, the line with the highest *IiUGT4* expression, had lariciresinol-4-*O*-β-D-glucoside, lariciresinol-4′-*O*-β-D-glucoside, and clemastanin B contents that were 2.52, 3.36, and 1.65 times as high as the controls.

In the *IiUGT71B5a*-OE lines, the coniferin content was significantly higher than in the control line. For example, line *IiUGT71B5aOE*-2 had the highest *IiUGT71B5a* expression and accumulated the most abundant coniferin (up to 40.06 mg⋅g^–1^ DW), which was 23.60 times as high as the control (1.69 mg⋅g^–1^DW). Although *IiUGT71B5a*-OE lines accumulated higher pinoresinol diglucoside content, the *IiUGT1*-OE lines accumulated much higher levels of pinoresinol diglucoside compared with the *IiUGT71B5a*-OE lines. The lariciresinol-4-*O*-β-D-glucoside and clemastanin B contents were higher in *IiUGT71B5a*-OE lines, which was similar to *IiUGT1* (Figure 7F). This indicates that both *IiUGT1* and *IiUGT71B5a* may be involved in the biosynthesis of lariciresinol glycosides and suggests that there was some functional redundancy amongst these three *IiUGT*.

### Molecular Docking and Site-Directed Mutagenesis of IiUGT4

**FIGURE 8:**
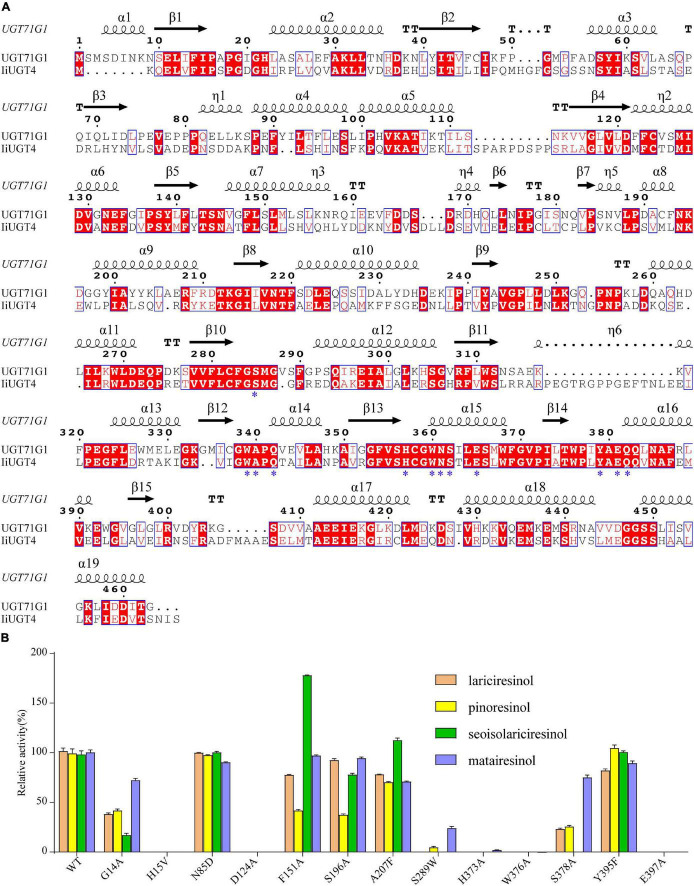
Structure-based sequence alignment and site-directed mutagenesis revealed the essential residues of IiUGT4. **(A)** Structure-based sequence alignment of IiUGT4 using UGT76G1 structure as a reference. α-helices was presented as large squiggles, β-strands as arrows, and strict β-turns as TT letters. The conserved residues interacting with the substrate were labeled with star symbols. **(B)** site-directed mutagenesis to identification of the essential residues of IiUGT4.

In this study, *IiUGT4* plays an important role in converting lariciresinol to the relevant glycosides in *I. indigotica*. Molecular modeling and site-directed mutagenesis were performed to further insight into the protein structure and key residues determining IiUGT4 reaction patterns. As shown in Figure 8A, the substrate UDP-glucose interacted with the same amino acid residues consistent with UGT71G1 in the PSPG motif. These key residues interacted with the uracil ring, the ribose ring, and the glucose ring of UDP-glucose in UGT71G1. The conserved catalytic dyad residues were His15/Asp124 in IiUGT4 consistent with His22/Asp121 in UGT71G1.

Site-directed mutagenesis and enzyme assays were performed to assess whether these IiUGT4 residues affect its enzymatic activity toward various substrates. The mutants in IiUGT4 to five residues (H15A, D124A, H373A, W376A, E397A) significantly decreased lignan glucosylation activity (Figure 8B). The glycosylation efficiency of F151A and S196A mutations generally decreased in the two substrates tested, while the F151A mutation selectively increased secoisolariciresinol activity by 78.39%. Additionally, A207 mutation catalytic activity selectively increased in secoisolariciresinol by 14.02%, but decreased in the other substrates. Compared with the wild-type enzyme, the S289W mutation retained little *O*-glycosylation of pinoresinol and matairesinol. These results demonstrated that H15, D124, H373, W376, and E397 play critical roles in the *O*-glycosylation of IiUGT4, and that F151 could be related to the substrate preference.

## Discussion

Several recent studies have assessed the biosynthetic pathway of lignan glycosides in *I. indigotica*, but the key *UGTs* that functions in the biosynthesis of anti-viral lignan glycosides such as lariciresinol-4-*O*-β-D-glucoside and clemastanin B have remained unclear (Hu et al., 2011; Li et al., 2014; Chen et al., 2015; Dong et al., 2015; Xiao et al., 2015; Ma et al., 2016; Zhang et al., 2016; Chen et al., 2021). Here, three UDP-glucose lignan glycosyltransferases from *I. indigotica* were identified efficiently catalyzing the glycosylation at the 4-hydroxyl group and 4′-hydroxyl group of lariciresinol *in vitro*. However, additional transgenic results have demonstrated that three *UGT* genes play specific roles in the glycosylation of lignan *in planta*: *IiUGT71B5a* and *IiUGT1* primarily participate in the biosynthesis of coniferin and pinoresinol diglucoside, respectively, while *IiUGT4* serves an important part during the biosynthesis of lariciresinol glycosides in *I. indigotica*. These results demonstrated that the three lignan-related *IiUGTs* play specific roles in the biosynthesis of lignan glycosides, and established the outlines of the biosynthetic route to lignan glycosides in *I. indigotica*.

Only a few lignan-related *UGTs* have been identified in plants and these enzymes exhibit distinct catalytic properties. For example, UGT71A9 prefers glycosylation of (+)-sesaminol, UGT94D1 specifically glycosylates (+)-sesaminol 2-*O*-glucoside, while UGT74S1 is speicfic for the glycosylation of (+)-secoisolariciresinol (Noguchi et al., 2008; Ghose et al., 2014). Although UGT71C1 was shown to have activity toward pinoresinol and lariciresinol, the products were not diglucosides compared with the IiUGTs (Atsushi et al., 2014). In this study, various kinds of carbon skeletons can be glycosylated by three IiUGTs enzymes, including furan (lariciresinol), furofuran (pinoresinol), dibenzylbutyrolactone (matairesinol), and dibenzylbutane (secoisolariciresinol). As same as previous studies, IiUGT71B5a showed adequate affinity to pinoresinol and matairesinol, however, IiUGT4 has a higher affinity for matairesinol and pinoresinol (Chen et al., 2021). Furthermore, IiUGT4 has the highest affinity for lariciresinol compared with IiUGT1 and IiUGT71B5a. These three enzymes have a comparative affinity to secoisolariciresinol, indicating that these IiUGTs exhibit enzyme promiscuity. These results indicate that these *IiUGTs* could play multiple roles by catalyzing the glycosylation of different substrates *in planta*.

The amino acid sequence of IiUGT4 was 61.81% similar to IiUGT71B5a, putting them in the same subfamily, UGT71B, and the results of an *in vitro* assay found that they shared similar functional behaviors, which suggesting that they originated from a similar ancestor and subsequently diverge. Their similar transcription patterns support this hypothesis. Previous studies have demonstrated that the UGT71 family plays multiple roles: catalyzing diverse substrates such as flavonoid/triterpene (*Medicago truncatula* UGT71G1) and lignan (UGT71A9 and UGT71A18),serving as a growth inhibitor (*A. thaliana* UGT71B2/HYR1), exogenous curcumin (*Catharanthus roseus* UGT71E2), an endogenous phytohormone (*A. thaliana* UGT71B6), and exogenous naphthols (*Nicotiana tabacum* UGT71A6, UGT71A7, UGT71A11) (Taguchi et al., 2001; Shao et al., 2005; Priest et al., 2006; Noguchi et al., 2008; Eiichiro et al., 2010; Le et al., 2016; Rehman et al., 2018). In this study, both IiUGT4 and IiUGT71B5a belong to the UGT71 family. Similar to reported lignan-related UGT71 family members (UGT71A9 and UGT71A18), these results also support the view that the UGT71 family enzymes possess promiscuous substrate specificity, some of which have adapted to lignan. The position of the PSPG conserved motif of the ORFs of the three *I. indigotica* IiUGTs was identified, which found they have a similar location to sesame UGT71A9. The sequence of the HCGW region of IiUGT1 was 70.45 and 52.27% similar to that of sesame lignan glycosylation UGT71A9 (XP_011100453.1) and flax lignan glycosylation UGT74S1 (AGD95005), respectively. The PSPG sequences of IiUGT4 and IiUGT71B5a were 75.51% similar to UGT71A9, but only 50.13 and 56.82% similar to UGT74S1, respectively (Supplementary Figure 11). The structural diversity of lignan glycosyltransferases strongly suggest that not all lignan UGTs belong to the UGT71 family, since flavonoid UGTs form separate phylogenetic clades based on their various regio-specificities (Yonekura-Sakakibara and Hanada, 2011). Based on the phylogenetic tree, IiUGT1 and UGT72E2 from *A. thaliana* are grouped within the same subfamily, UGT72E (Lanot et al., 2006). However, the catalytic characteristics of IiUGT1 were different from UGT72E2. Unlike the monolignol-specific UGT72E family members from *A. thaliana*, IiUGT1 can catalyze monolignol and other lignans into their corresponding glycosides. In the model plant *A. thaliana*, UGT72E1, UGT72E2, and UGT72E3 were monolignol-specific UGTs. During a preliminary screening, the lignan activity of all four UGT72E subfamily members (IiUGT1, IiUGT3, and IiUGT9) from *I. indigotica* was tested. Only one of them, IiUGT1, demonstrated catalytic activity toward lariciresinol and pinoresinol diglucosides (Supplementary Figures 12–15). These results suggest that some functional diversification occurred in the UGT72E subfamily of *I. indigotica*.

Our RNA interference experiments demonstrated that *IiUGT71B5a* and *IiUGT1* were primarily participating in the biosynthesis of coniferin and pinoresinol diglucoside, respectively. *IiUGT4* was primarily responsible for the glycosylation of lariciresinol. Lariciresinol-4′-*O*-β-D-glucoside content reduced as *IiUGT71B5a* transcription decreased, suggesting that *IiUGT71B5a* performed similar functions to other lignan-related *IiUGTs*. This could explain why the lariciresinol glycosides contents increased as transcript levels of *IiUGT71B5a* increased in *IiUGT1*-RNAi lines. It is noteworthy that *IiUGT71B5a*, previously studied showing a preference for pinoresinol *in vitro* (Chen et al., 2021), was functioning as a coniferyl alcohol glycosyltransferase *in vivo*. This phenomenon also occurred in other plant UGTs such as UGT78G1 and UGT73C6, whose activities *in vivo* were not consistent with their *in vitro*, indicating that it is necessary to validate the roles of UGTs *in planta* (Peel et al., 2009; Yin et al., 2017). These *IiUGTs* could be the primary enzymes involved in lignan glycosylation, making it important to determine the consequences of their overexpression in *I. indigotica* and their potential implications in metabolic engineering. As expected, the pinoresinol diglucoside content significantly increased following introduction of *IiUGT1* gene, in addition, the contents of lariciresinol-4-*O*-β-D-glucoside and lariciresinol-4′-*O*-β-D-glucoside also significantly increased (Figure 7B). This strongly indicates that IiUGT1 could play other roles in the lignan glycosylation of *I. indigotica* and could be explained by the catalytic activities of multiple lignans *in vitro*. Functional redundancies across these three *IiUGTs* could explain why *IiUGT71B5a*-OE lines have higher accumulated contents of pinoresinol diglucoside and lariciresinol glycosides. These results suggest that both *IiUGT1* and *IiUGT71B5a* are involved in the biosynthesis of lariciresinol glycosides and exhibit functional redundancy. Only the lariciresinol glycosides content significantly increased following introduction of *IiUGT4* gene, it indicates that the dominant role of *IiUGT4* is to glycosylate lariciresinol in *I. indigotica*. These results demonstrated that the three lignan-related IiUGTs play specific roles in the biosynthesis of lignan glycosides and highlight the importance of efficiently producing these lignan glycosides *via* gene-mediated metabolic engineering (Supplementary Figure 16).

The results from both *in vitro* assay and analysis of transgenic hairy roots indicate that *IiUGT4* plays a critical role in the biosynthesis of lariciresinol glycosides in *I. indigotica*. Molecular docking and site-directed mutagenesis demonstrated that H373, W376, and E397 were key residues for catalytic activity, and that the F151 site was related to the substrate selectivity. As shown in Supplementary Figure 17, a hydrogen bond formed between the furan ring and the W376 site of IiUGT4, strongly suggests that the furan ring was critical for specific recognition by IiUGT4. The site-direct mutagenesis further supports this hypothesis (Figure 8). Similar to W376, the H373 site was linked to lariciresinol by a conventional H-bond. When F151 was muted to A, the recombinant protein displayed higher activity against secoisolariciresinol, suggesting that this site was a potential target for modifying its substrate preference by site-mutagenesis. The strict conservation of H15 and D124 sites across plant UGTs indicates that they were critical for IiUGT4 enzyme activity by binding with sugar donors. Both H15A and D124A ceased catalytic activities toward tested lignans, which was consistent with previously published literature (Shao et al., 2005; Zhang et al., 2020). Our analysis of IiUGT4 revealed five key residues for its catalytic activity, and F151 could be associated with the substrate preference, and provides the foundation for further revealed the catalytic mechanism of IiUGT4.

Along with the identification of three lignan-related *IiUGTs* from *I. indigotica*, the most important part of the lignan glycosylation map has been discovered: *IiUGT71B5a* and *IiUGT1* are primarily participating in the biosynthesis of coniferin and pinoresinol diglucoside, respectively. *IiUGT4* is primarily responsible for the glycosylation of lariciresinol. The identification of these genes highlights the possibilities for engineering a lignan-related synthetic pathway using heterologous plant systems or microorganisms.

## Data Availability Statement

The datasets presented in this study can be found in online repositories. The names of the repository/repositories and accession number(s) can be found in the article/Supplementary Material. UGT committee has assigned names UGT72E8, UGT71B18, UGT71B19 to IiUGT1, IiUGT4, and IiUGT71B5a, respectively.

## Author Contributions

YT: conceptualization, methodology, and original draft preparation. JY: methodology and NMR analysis. YJ, JW, YL, YZ, and BJ: methodology, software, and visualization. XW: molecular modeling. TC: bioinformatics analysis. LK: chemical analysis. JG and GC: data analysis and reviewing. JT: investigation, validation, writing reviewing, supervision, and editing. LH: funding acquisition, project administration, and resources.

## Conflict of Interest

The authors declare that the research was conducted in the absence of any commercial or financial relationships that could be construed as a potential conflict of interest.

## Publisher’s Note

All claims expressed in this article are solely those of the authors and do not necessarily represent those of their affiliated organizations, or those of the publisher, the editors and the reviewers. Any product that may be evaluated in this article, or claim that may be made by its manufacturer, is not guaranteed or endorsed by the publisher.

## Footnotes

1
www.ExPASy.org

2
https://swissmodel.expasy.org/
