# Supplementary material for: Functional Characterization of UDP-Glycosyltransferases Involved in Anti-viral Lignan Glycosides Biosynthesis in Isatis indigotica
Source: Front Plant Sci. 2022 Jun 14;13:921815. doi: 10.3389/fpls.2022.921815 (PMC9237620; doi:10.3389/fpls.2022.921815)
Supplement: Supplementary file 1 [file Data_Sheet_1.PDF]

Supplementary Material

Functional Characterization Of UDP-glycosyltransferases Involved in Anti-viral Lignan Glycosides Biosynthesis in *Isatis indigotica*

Yuping Tan[1,2], Jian Yang[2], Yinyin Jiang[2], Jian Wang[2], Yahui Liu[3], Yujun Zhao[2], Baolong Jin[2], Xing Wang[4, 5],Tong Chen[2], Liping Kang[2], Juan Guo[2], Guanghong Cui[2], Jinfu Tang[2, *], Luqi Huang[1, 2,*]

[1]School of Traditional Chinese Medicine, Shenyang Pharmaceutical University, Shenyang, PR China

[2]State Key Laboratory of Dao-di Herbs, National Resource Center for Chinese Materia Medica, China Academy of Chinese Medical Sciences, Beijing, PR China

[3]National Institute of Metrology, Beijing, PR China

[4]School of Traditional Chinese Medicine, Capital Medical University, Beijing, PR China

[5]Beijing Key Lab of TCM Collateral Disease Theory Research, Capital Medical University, Beijing, PR China

*** Correspondence:**

Jinfu Tang
jinfutang@126.com

Luqi Huang
huangluqi01@126.com

1 Supplementary Figures and Tables

1.1 Supplementary Figures

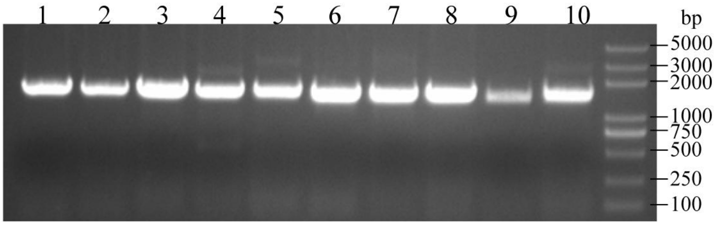

Supplementary Figure 1. PCR amplifiation of the ORF of ten candidate *IiUGT* genes.

Lane M: DNA Marker DL5000; Lane 1~Lane 10: *IiUGT1~IiUGT71B5a*.

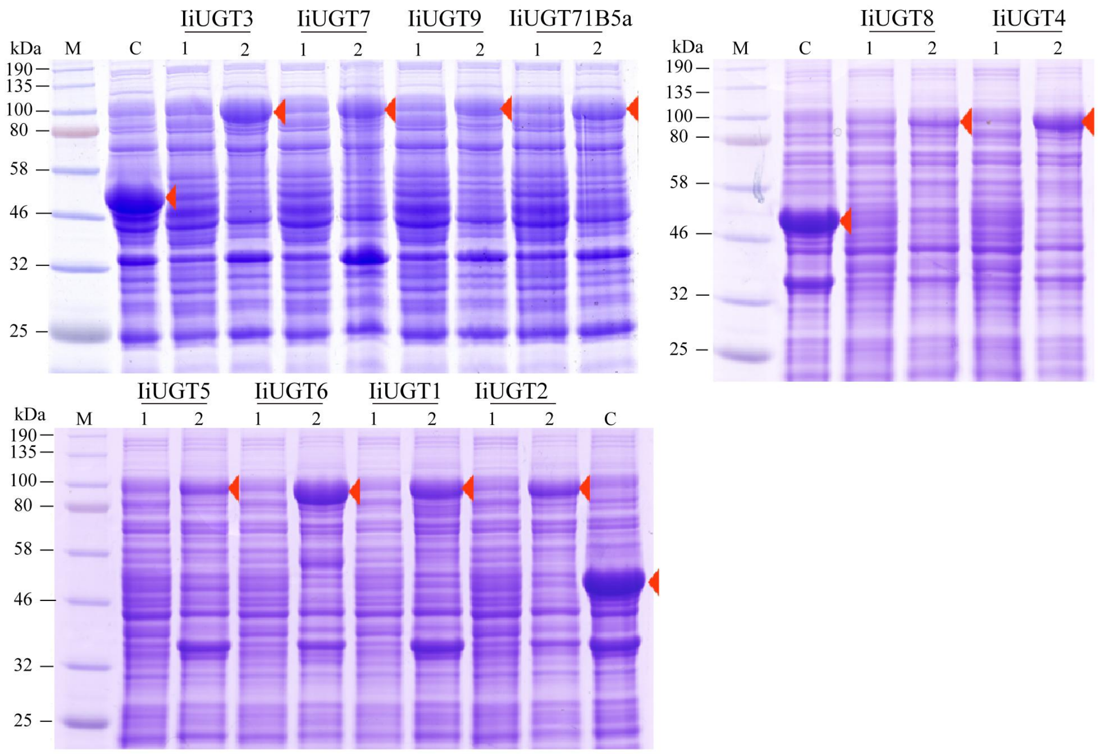

Supplementary Figure 2. SDS-PAGE analysis of expression of His-MBP-pET28a-*IiUGT*.

Lane M: molecular weight marker; Lane C: The crude protein induced by IPTG in *E. coli* Rosetta (DE3) containing the empty vector induced by IPTG; Lane 1: The crude protein in *E. coli* Rosetta (DE3) containing the recombinant vector without IPTG; Lane 2: The crude protein in *E. coli* Rosetta (DE3) containing the recombinant vector induced by IPTG.

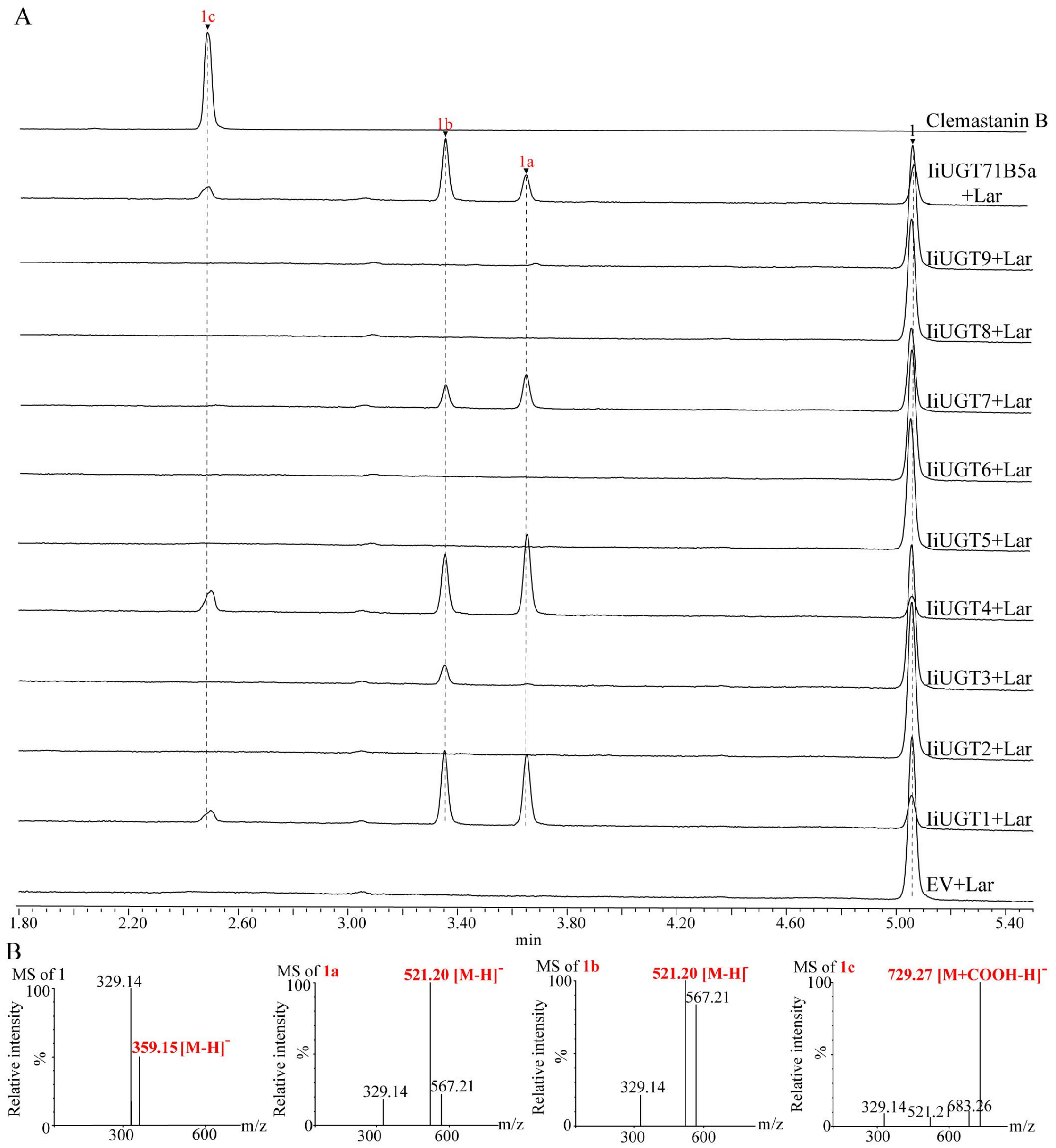

Supplementary Figure 3. UPLC/Q-TOF-MS analysis of the reactions of candidate IiUGTs with UDP-glucose and (1) lariciresinol as substrates.

(A) The enzyme reactions of crude proteins of *E. coil* carrying empty vector (EV), IiUGTs were assayed with UDP-glucose as the sugar donor. (1) lariciresinol; (1a) lariciresinol-4'-*O*-*β*-D-glucoside; (1b) lariciresinol-4-*O*-*β*-D-glucoside; (1c) clemastanin B. (B) MS spectra of the products in negative mode.

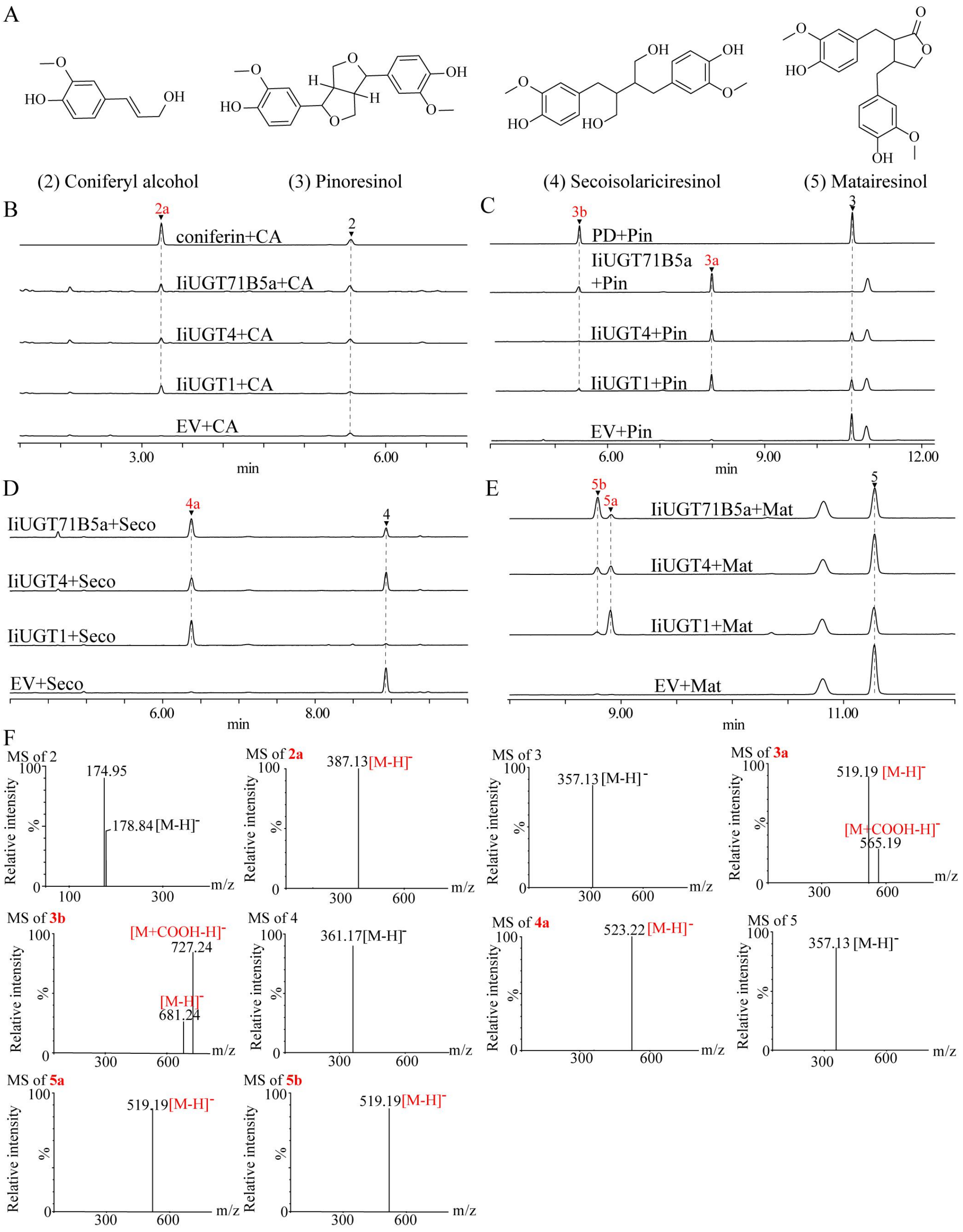

Supplementary Figure 4. Enzymatic activity of candidate IiUGT proteins.

(A) Chemical structures of lignans (glycosy acceptors). UPLC-ESI-MS/MS analysis of candidate IiUGT enzymatic reaction products against coniferyl alcohol (B) and pinoresinol (C), secoisolariciresinol (D) and matairesinol (E), respectively. The enzyme reactions of crude proteins of *E. coil* carrying empty vector (EV), IiUGT1, IiUGT4, IiUGT10 were assayed with UDP-glucose as the sugar donor. (B) 2. CA, coniferyl alcohol; 2a. coniferin. (C) 3. Pin, pinoresinol; 3b. PD, pinoresinol diglucoside. (D) 4. Seco, secoisolariciresinol. (E) 5. Mat, matairesinol. (F) MS spectra of the products in negative mode.

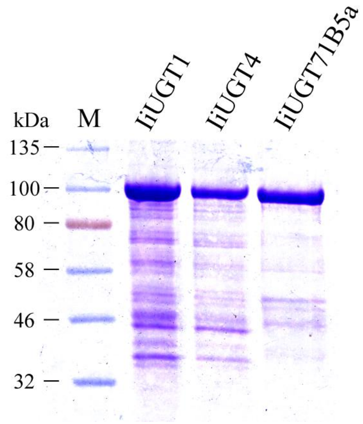

Suppelmentary Figure 5. SDS-PAGE analysis of purified proteins.

SDS-PAGE analysis showed that the purified IiUGTs fused with a HIS-MBP tag (41.34 kDa).

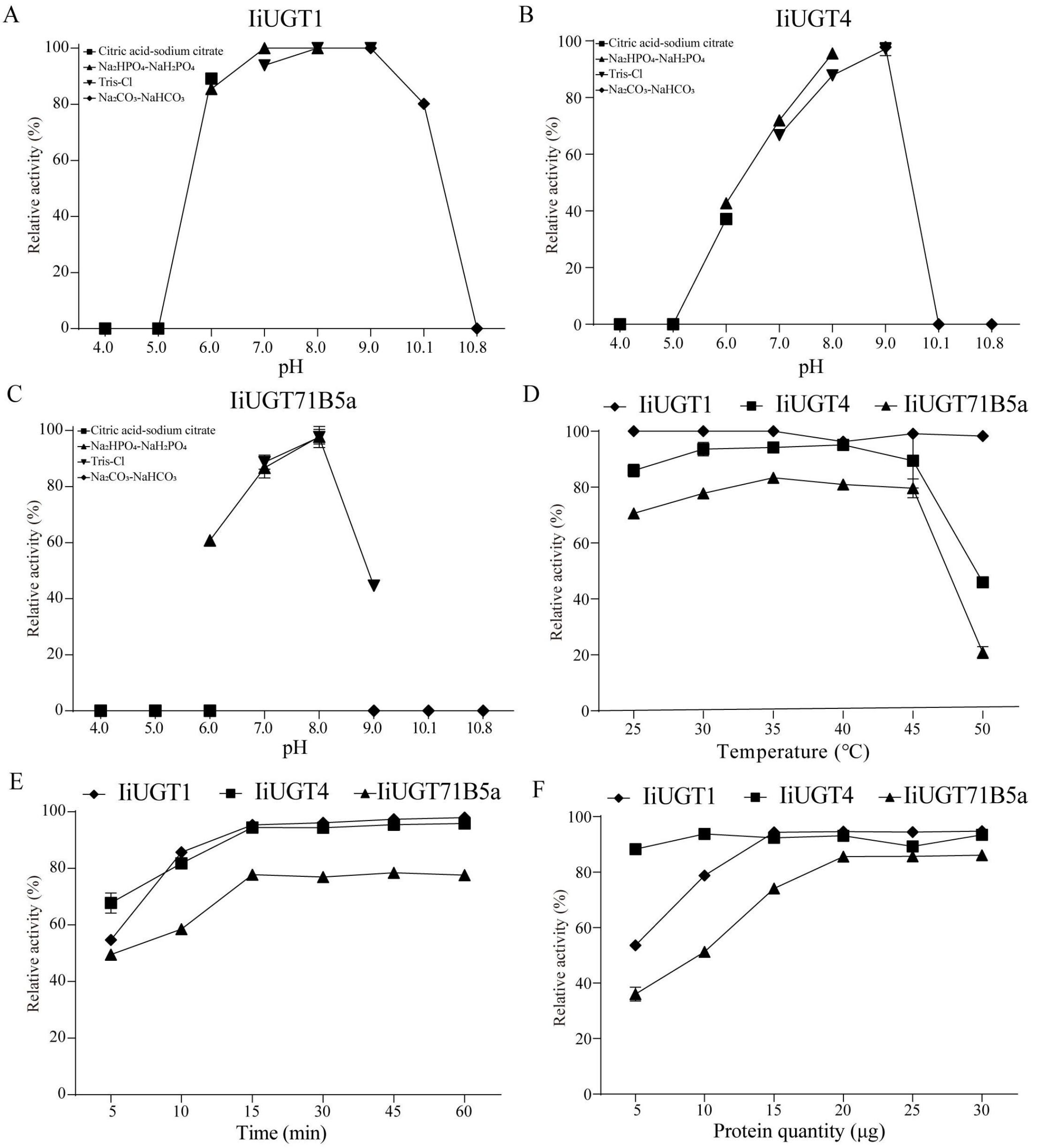

Supplementary Figure 6. Properties of recombinant IiUGTs.

To verify pH prefenrence (A), (B), (C), optimal temperature (D), optimal reactive time (E), and optimal reactive protein quantity (F), reactions were examined using lariciresinol ans UDP-glucose as substrates as described in the experimental procedures. The squares represent Citric acid-sodium citrate Buffer (pH 4.0-6.0), the triangles represent Sodium phosphate Buffer (pH 6.0-8.0), the inverted triangles represent Tris-Cl Buffer (pH 7.0-9.0) and the diamonds represent Sodium carbnote Buffer (pH 9.0-10.8) in (A), (B), (C). Values of the relative activities are average ± SD (n=3), with maximum activity levels assumed to be 100%.

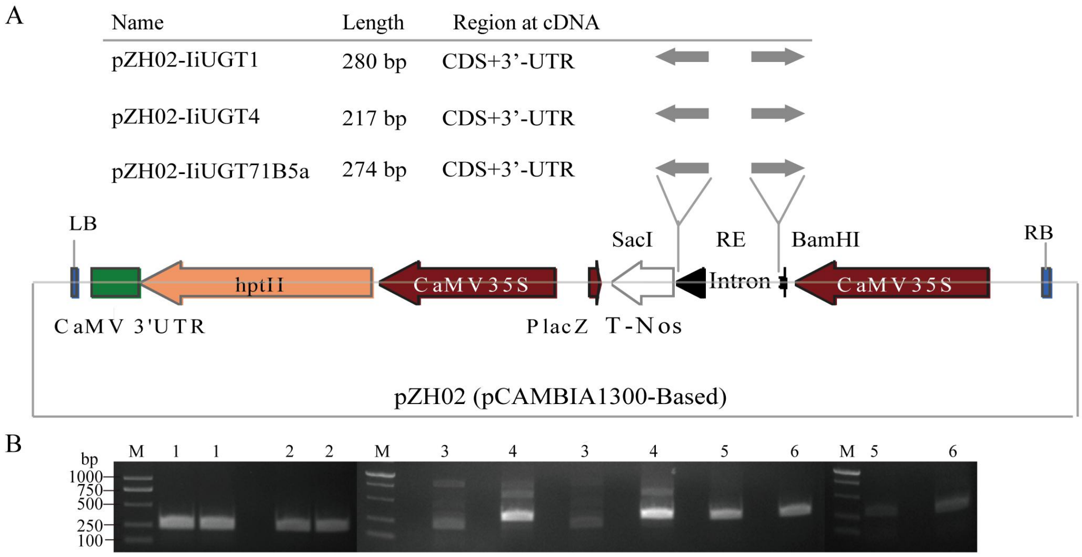

SUpplementary Figure 7. RNAi silencing of *IiUGT1*, ***IiUGT4*** and *IiUGT10* in *I. indigotica* hairy roots.

(A) Schematic diagram of the RNAi vector. CaMV 35S, CaMV 35S promater; hpt II, hygromycin resistance gene; Intron, the Pdk intron; grey arrow, the 200-300bp fragment of the coding region of *IiUGTs*. (B) PCR amplifiation of the target *IiUGT*s interference fragmnets. M: DNA Marker; Lane 1: *IiUGT4*-RNAi sence; Lane 2: *IiUGT4*-RNAi antisence; Lane 3: *IiUGT10*-RNAi sence; Lane 4: *IiUGT1*-RNAi sence; Lane 5: *IiUGT1*-RNAi antisence; Lane 6: *IiUGT10*-RNAi antisence.

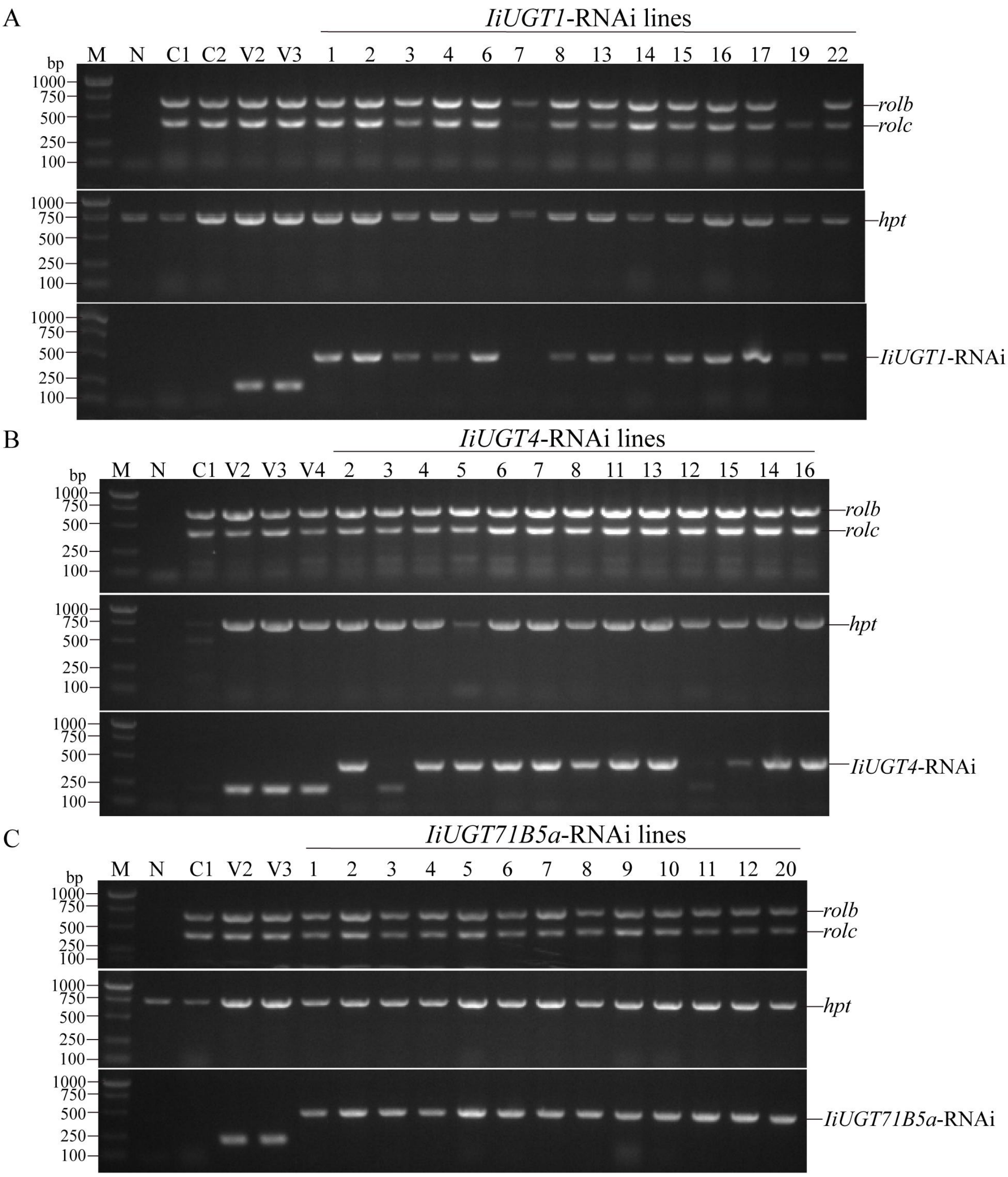

Supplementary Figure 8. PCR analysis of *rolb*, *rolc*, *hpt* and additional *IiUGT*-RNAi fragment in *I. indigotica* hairy roots.

Lane M: DNA Marker; Lane N: Negative control; Lane C1, C2: Wild-type lines; Lane V2, V3: Control lines with the empty construct.

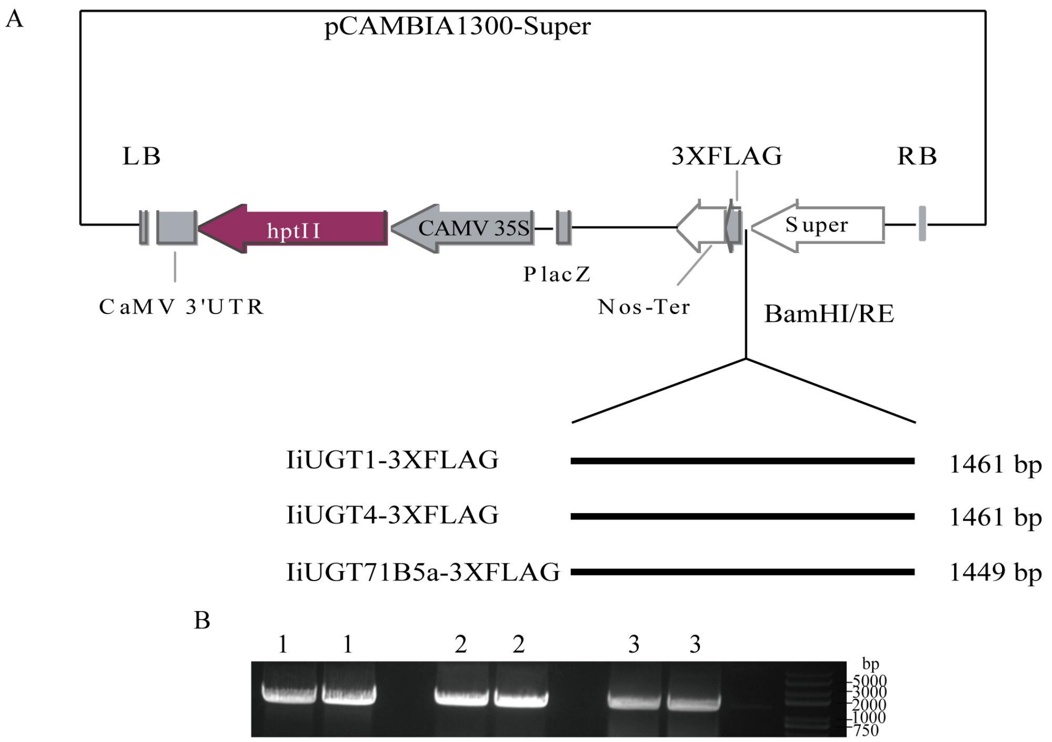

Supplementary Figure 9. Overexpression of *IiUGTs* in *I. indigotica* hairy roots.

(A) The full length ORF of *IiUGTs* were inserted into the pCAMBIA1300-Super expression vector under control of the Super promoter, respectively. (B) PCR amplification of the ORF of the target *IiUGT*s. Lane M: DNA Marker; Lane 1: *IiUGT1*; Lane 2: *IiUGT4*; Lane 3: *IiUGT10*.

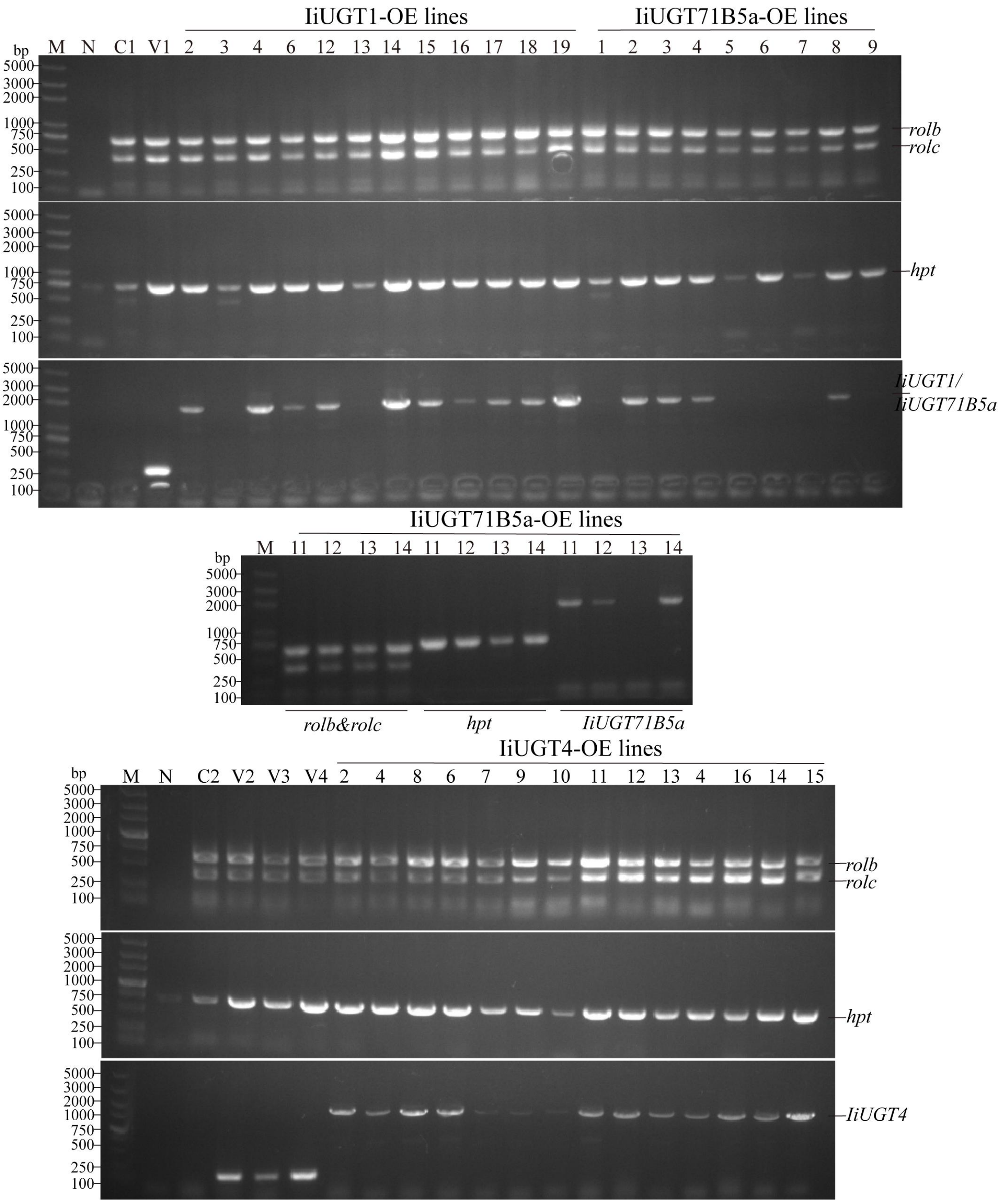

Supplementary Figure 10. PCR analysis of *rolb*, *rolc*, *hpt* and the exogenous *IiUGT* gene with flag tag in *I. indigotica* hairy roots.

(A) Lane M: DNA Marker; Lane N: Negative control; Lane C1, C2: Wild-type lines; Lane V1, V2, V3: Control lines with the empty construct.

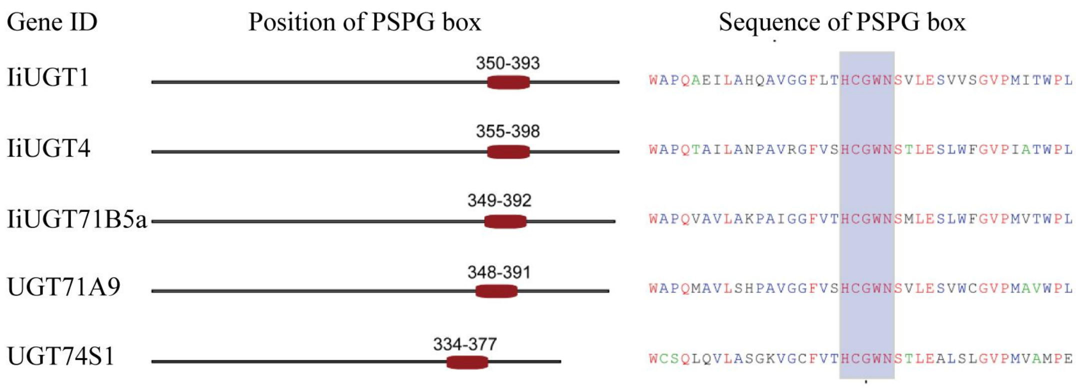

Supplementary Figure 11. Amino acid sequence alignment of the PSPG conserved motif for IiUGTs and lignan glycosylation UGTs.

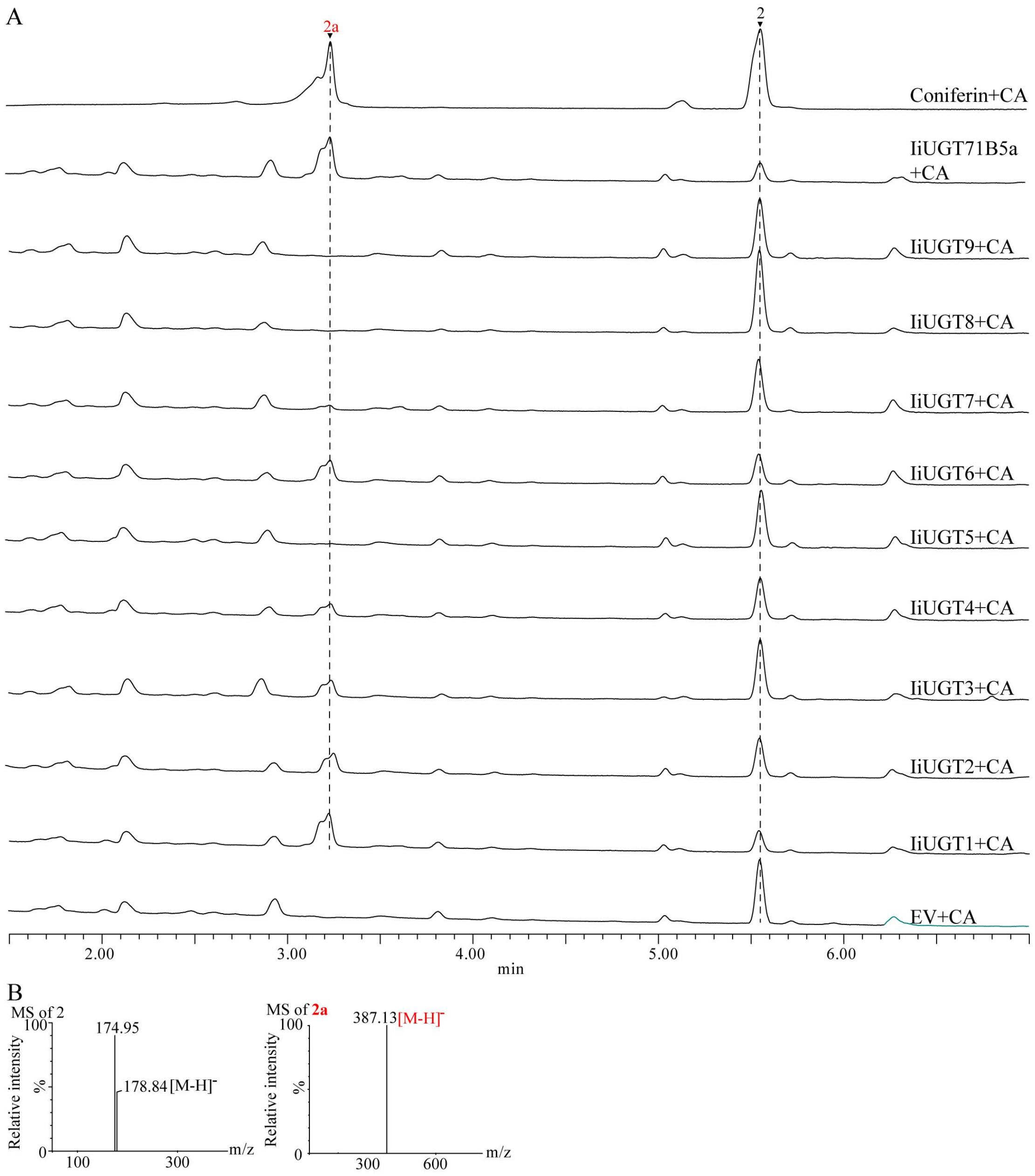

Supplementary Figure 12. UPLC/Q-TOF-MS analysis of the reactions of candidate IiUGTs with UDP-glucose and (2) coniferly alcohol as substrates.

(A) The enzyme reactions of crude proteins of *E. coil* carrying empty vector (EV), IiUGTs were assayed with UDP-glucose as the sugar donor. CA, coniferyl alcohol. (B) MS spectra of the products in negative mode.

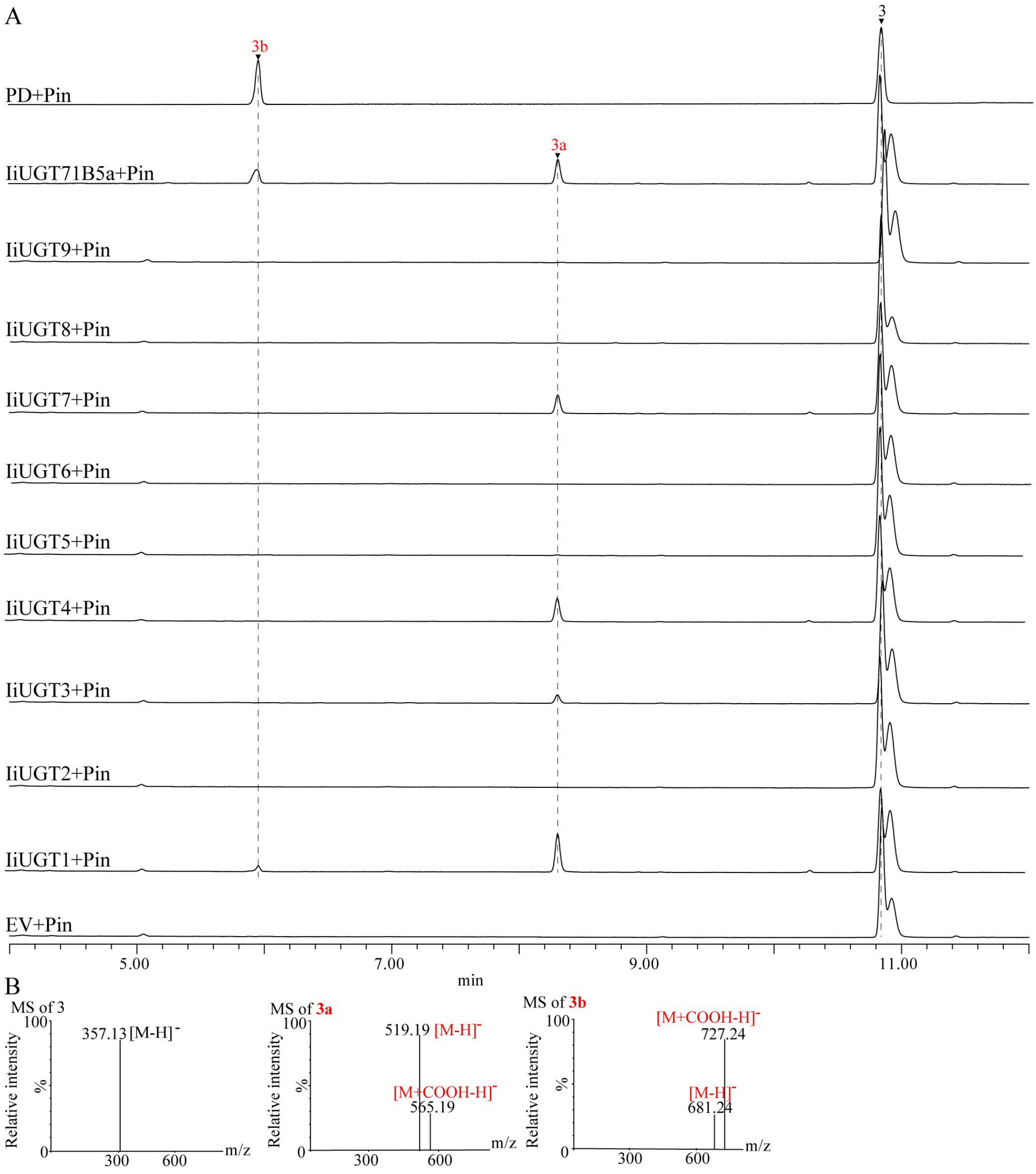

Supplementary Figure 13. UPLC/Q-TOF-MS analysis of the reactions of candidate IiUGTs with UDP-glucose and (3) pinoresinol as substrates.

The enzyme reactions of crude proteins of *E. coil* carrying empty vector (EV), IiUGTs were assayed with UDP-glucose as the sugar donor. Pin, pinoresinol; PD, pinoresinol diglucoside.

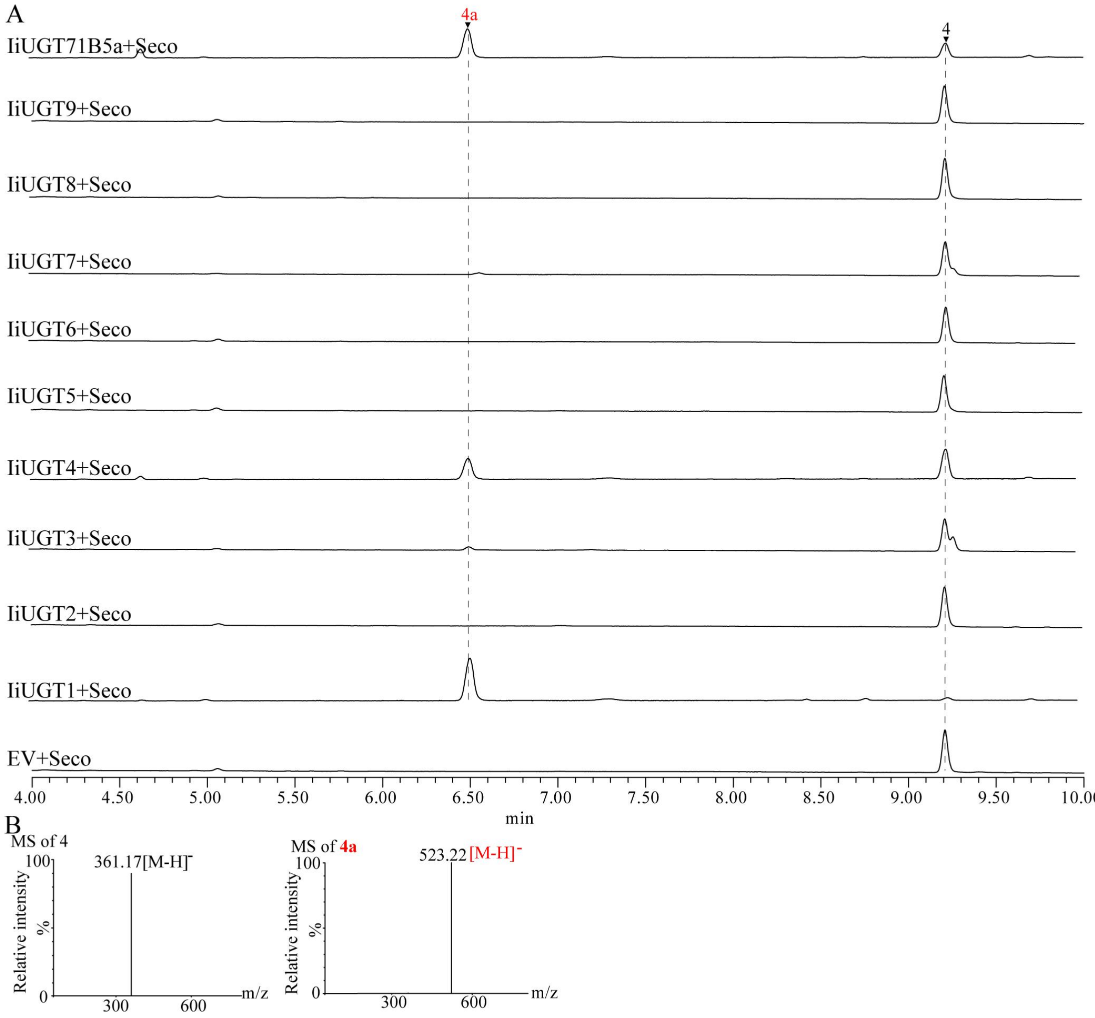

Supplementary Figure 14. UPLC/Q-TOF-MS analysis of the reactions of candidate IiUGTs with UDP-glucose and (4) secoisolariciresinol as substrates.

The enzyme reactions of crude proteins of *E. coil* carrying empty vector (EV), IiUGTs were assayed with UDP-glucose as the sugar donor. Seco, secoisolariciresinol.

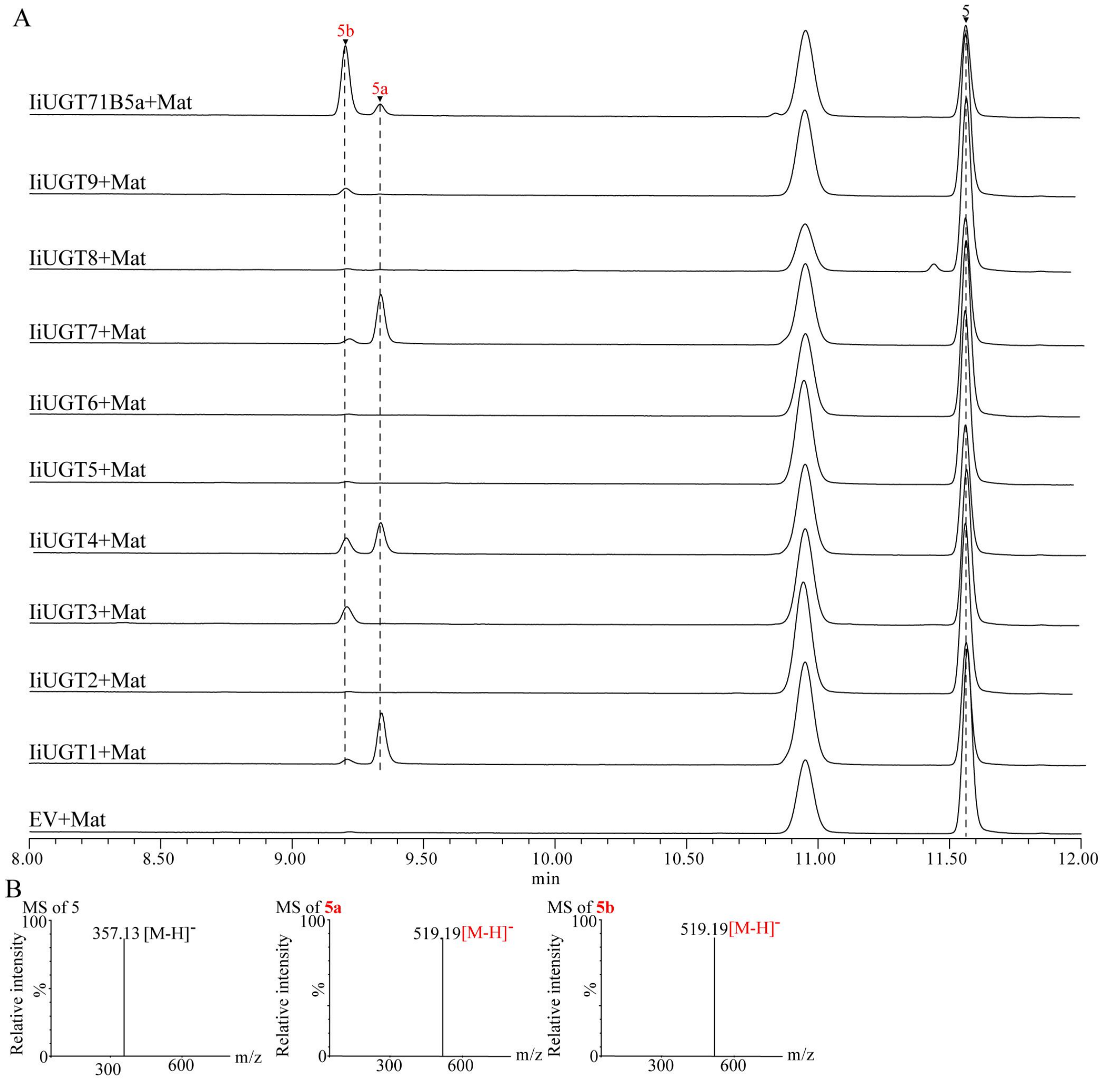

Supplementary Figure 15. UPLC/Q-TOF-MS analysis of the reactions of candidate IiUGTs with UDP-glucose and (5) matairesinol as substrates.

The enzyme reactions of crude proteins of *E. coil* carrying empty vector (EV), IiUGTs were assayed with UDP-glucose as the sugar donor. Mat, matairesinol.

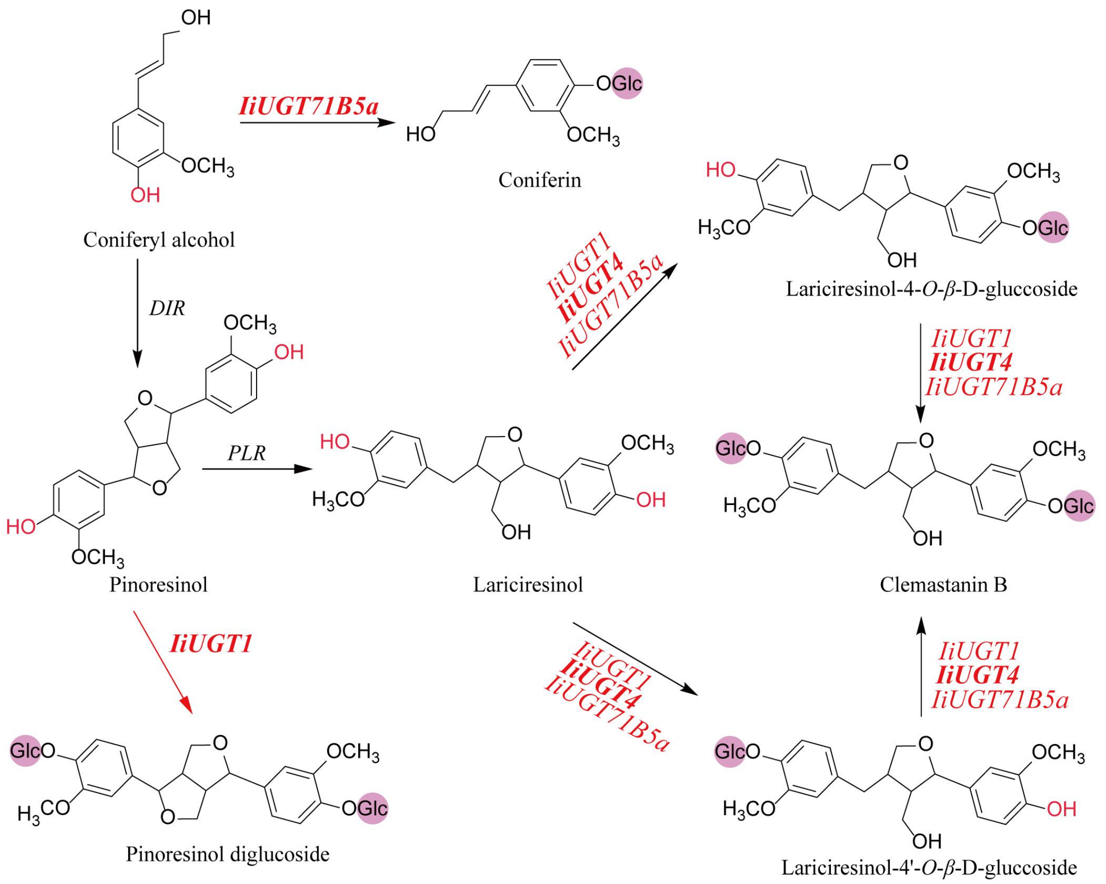

Supplementary Figure 16. The deductive pathway of lignan glycosides in *I. indigotica*.

IiUGT genes playing a dominant role in the biosynthesis were in red blod.

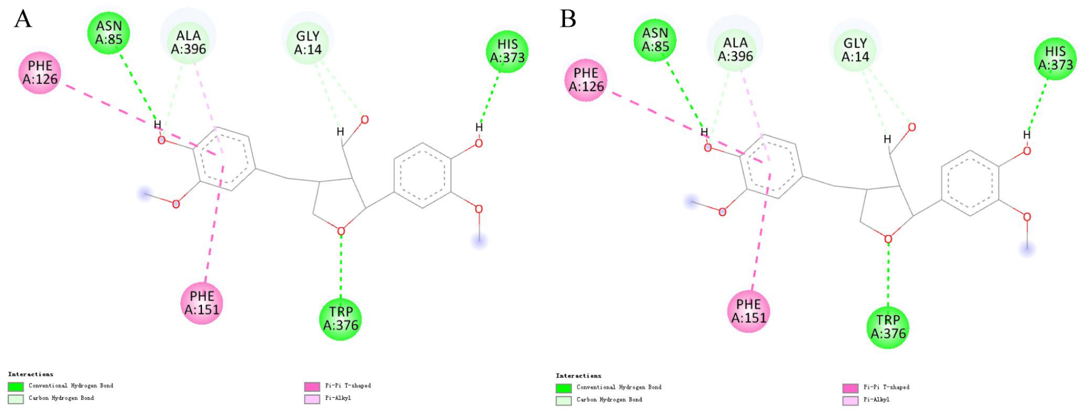

Supplementary Figure 17. Molecular docking of IiUGT4 with (+)-lariciresinol (A), (-)-lariciresinol (B).

1.2 Supplementary Tables

Supplementary Table 1. Unigenes generated from *I. indigotica* transcriptome.

Range of Length（bp）	Total Number	Percentage（%）
200~300	19,709	33.43
300~500	13,458	23.58
500~1000	10,032	17.58
1000~2000	8,646	15.15
>2000	5,857	10.26
Total	57,072	100
Total Length	47,238,106	--
N50	1,546	--
Mean	827.69	--

Supplementary Table 2. The 10 *IiUGT* genes cloned in the study.

Gene Name	Subfamily	Protein Squences
IiUGT1	*UGT72E*	mkitrphavmfaspgmghvipvielgkrlvgshgfqvtifvleadaasaqsq<br>flnstgcdatlidviclptpdisglvdpsaffaiklltmmretiptirskieemqh<br>kptalivdlfgldalrlggefnmltyvfiasnarfvaltlyfptlekdaeeehiikk<br>kplampgcepvrfedtlepfldptdqiyrifvpfglvyptadgiivntwddme<br>pktlkslqdpkllgriarvpvypigplsrpvdpsktnhpvldwlnkqpeesvl<br>yisfgsggslsakqltelawglelsqqrfvwvvrppvdssacseylsansgev<br>qdgtpdylpkefisrtqerglvvpswapqaeilahqavggflthcgwnsvles<br>vvsgvpmitwplfadqkmnatllneelgvairsrklpseevtlrveieslvrrl<br>mvedegremrekvkklrdtaemslrcdggsheslsrvanechrllerarma<br>rga
IiUGT2	*UGT71C*	maketelifipvpstghllvniefakrlinlehriqtitiiqmdspinphasvfars<br>lvasqpqirlhslpvlhdpppsdlykrapeayivqlvkkttplvkdavssivesr<br>gsdsvrvaglvldffcnslikdvgnelnlptyifltcnarylsmmkyipdrhrk<br>maskldwssgdeelpipgfanpiptkfmppglfnqegyeayvelaprfaha<br>kgilvnsiaelephpfgyfsqqhnyppvypvgpilslkdraspneeeaadrdr<br>ilrwledqpessvvflcfgskgsvdepqvkeiaqalevvgcrflwsirmsleei<br>kpsdvlpegfmgrvagrglvcgwapqvevlahkaiggfvshcgwnstlesl<br>wfgvpvatwpmyaeqqlnaftlvkelglavdlrmdyvsgrgglvtcdeiara<br>vrslmdggegkrvkvkemadaarkammdggssylatarfigellddgss
IiUGT3	*UGT72E*	mqitkphaamfsspgmghlipvielakrlsanhgfrvtvfvlesdaasaqskf<br>lnstgvdvvnlpspdisdlvdpadhvvtkigiimreavpalrskiaemnqkpt<br>aliidlfgtdalclaaefkmltyvliasnarylgvamyyptldkhvqeehtvqrk<br>plevpgcepvrfedtmdaylvpdeplyrdfvrhslaypkadgilvntwdem<br>epkslkslqdpkllgrvarvpvypvgplcrpveqsktghpvldwlneqpdes<br>vlyisfgsggsltakqltelawgleqsqqrfvwvvrppvdgsscceyfsanga<br>ekkdstpeylpegfvtrtcdrglvvqswapqaevlahravggflthcgwnstl<br>egvvsgvpmiawplfaeqnmnaallsdelgiavradnlkeavtrfeieaivrk<br>vmteeegeemrmkvkklkdkaemllssdgggsaheslckvtvecerflerd<br>mdlarga
IiUGT4	*UGT71B*	mkqelvfipspgdghirplvqvakllvdrdehisitiliipqmhgfgsgssnsy<br>iaslstasedrlhynvlsvadepnsddakpnflshinsfkpqvkatveklitspa<br>rpdsppsrlagivvdmfctdmidvanefdvpsymfytsnatflgllshvqhly<br>ddknydvsdlldsevteleipcltcplpvkclpsvmlnkewlpialsqvrryke<br>tkgilvntfaelepqamkffsgednllptvypvgpilnlktngpnpaddkqsei<br>lrwldeqpretvvflcfgsmggfredqakeiaialersghrfvwslrrarpegtr<br>gppgeftnleeilpegfldrtakigkvigwapqtailanpavrgfvshcgwnst<br>leslwfgvpiatwplyaeqqvnafemveelglaveirnsfradfmaaeselm<br>taeeiergirclmeqdnvrdrvkemsekshvslmeggsshaallkfiedvtsn<br>is
IiUGT5	*UGT71D*	mivvlvntrkvrggnnrsyqflemrnaelifipaptvghlvpslelarrlidqdd<br>riritvlvmklqgqshldtyvksigsslpfvrfidvpeledkptfgstqsaeafvy

		dfierniplvrnivmdilsspaldgvtvkgivadffclpmvdvakdvslpfhvf<br>lttnsvflammqyladrhskdtsvfvrnsgemlsipgfvnpvpanvlptalfm<br>edgyeayvklailfakakgilvntyfdlepislnhfhneqnypsvyavgpvfn<br>pnaqphpdqdlarrdelvkwlddqpeasvvflcfgsmgrlsgplvkeiahgl<br>elcqyrflwslrteevthddlfpegfldrvsgrgmicgwspqveilahkavgsf<br>vshcgwnslveslwfgvpivtwpmyaeqqlnaflmvkelnlavemkldyr<br>vrsddlvnaneietaircvmnednnlvrkrvidisqmarkatlnggssylatek<br>fiqdvigikp
IiUGT6	*UGT71C*	mktselifvplpetghllstiefgkrlldldrrismitilsmklpyaphadaslaslt<br>asepgirlislpeiqdpppiklldtssetyildfveknipflrktirdlvsssgedsn<br>hvaglildffcvdlidigrevnlpsyifmtsnfgflgflqylperhrsissefdess<br>gdeelpipafvnrvpakvlppgvfdklsygtlvkigerlneakgifvnsfseve<br>pyaaehfsrggdypraypvgpvlnltgrtnpglasaqyaemmkwlddqpd<br>ssvlflcfgsmgvfsaaqiteiahaielvgfrfiwairtnmegdgdpheplpeg<br>fvdrtmgrgivcswapqvdilahkatggfvshcgwnsiqeslwygvpiatw<br>pmyaeqqlnafemvkelglaveirldyvadgdrvtleivsadeiaaairslmd<br>gdnlirkkvrevsaaarkavsdggssmvatgdfirdilgdhf
IiUGT7	*UGT72D*	meqphallvaspglghlipilelgnrlssvlnihvtvlavpsgssssptetea ira<br>avargtceiaelpsvdiehlvepdatvatrifekmratrpavqdavkamnrkp<br>tvmivdffatglmsvaddvgvtakyvyvpshawflavmvylpvldkvveg<br>eyidikepmkipgcrsvgpdelmdtmfdrsdrqyrecvrcgeeipmsdgil<br>vntweelqgntlaalredgelsrvmkvpvyaigpfvrsngpiekpksifewld<br>kqrdrsvvyvclgsggilsleqtmelawglelsgqsflwvlrrptsylearssdd<br>dqvsaglpegfldrtrgvglvvtqwapqveilshgsiggflshcgwssvleslt<br>kgvpivawplyaeqwmnatllteeigvavrtselpskkvigreevaslvrkiv<br>aeedeegrkvrakaeevkatseaawaqggsshgsllewakrcrlvcdsqii
IiUGT8	*UGT71C*	mgnqeiifvpypipghllvtielakylikrdnrihtitilhwtlplaphadlfaksl<br>vaseprirlftlpdvpnpppfelflrateayvleftkctvplvrealstivssrdgsd<br>pvwvaglvldffcvplievgnefnlpsyifltcnagflgilkylperhrriaselel<br>seehhpipgfvssvpskvlpsgqfvresyeawieiaqkfpkakgilvnsftcle<br>qnafdyfaclpenfppvypvgpvlsledrpspdldtsdqcrvmtwlddqpes<br>svvylcfgsfgvlgepqieeiaraleisshrflwsirtekatpydllpegfmdrtv<br>skglvcgwapqvevlahkavggfvshcgwnsvleslwfdvpiatwplyae<br>qqlnaytmvkelglsvelrldyvsakkvivkadeiagairslmdgedtprrrv<br>kkmaeaarmalmeggssfvavkrfiddlvgedf
IiUGT9	*UGT72E*	mqntkphaamltspgmghvipviqlgkrlagfhgfhvtifvleadaasaqsq<br>flnspgcdattlvdviglpspdisglvepsasfgtklltmmreavpsirskiaem<br>qhkptalivdllsldalrlggefnmltylfiasnarfvalmmyfptldrdveeeh<br>iikkkplaipgceplrfedtfeifldpssqmyqecvplglvyatvdgiivntwd<br>dmepktlkslqdpkllgriarvpiypigplcrpvdpsktnhpvldwlnkqpd<br>esvlyisfgsggslsakqltelawglelsqqrfvwvvrppvdgsacsayfsvnt<br>gqvrdgtpdylpeefvsrtlerglvvpswapqaeilahtavggflthcgwnsil<br>esvvigvpmiawplfaeqmtnatllneelgiavrsrrlpseglilreeiealvrri<br>mvdeegcvmrkkvkklsdtaekslscegtlsrvaeecerrlehdrsmarga

IiUGT71B5a	*UGT71B*	mkielvfipspgighlrstvelakqlvngddrlsitviiiprssggdatdsaqissl<br>faasqdrlryetisvadeptadrlptqlyiknqkpqvrdavakildparvdsppr<br>lagfvvdmfctsmidladefgvptymvytsnatflgitlhlqlmldekkydts<br>eldesvnelefpcltrpypveclpylfiskewlpffmdqarsfrkmkgilvntv<br>aelephalelfsgdddlpraypvgpvlhlesgsdnsnddgkqseilrwlddqp<br>aksvvflcfgsmggfneeqtreiavaldrsghrflwslrraspdilkqgpgdyt<br>dldevlpegfldrtlergkiigwapqaavlakpaiggfvthcgwnsmleslwf<br>gvpmvtwplyaeqkvnafvmveelglaveirrslkgdlmaggmetvaaed<br>ierairrvmeqdsdvrnrvkemaekchvaltdggssqvalrkfiqdvienvv<br>v

Supplementary Table 3.. GenBank accession numbers of UGT proteins in Figure 3.

Gene	Accession	Species
UGT71A5	BAF96584.1	*Antirrhinum majus*
UGT88D3	Q33DV3.1	*A. majus*
At3Rt	NP_564357.1	*Arabidopsis thaliana*
UGT71B1	NP_188812.1	*A. thaliana*
UGT71B6	NP_188815.2	*A. thaliana*
UGT71C1	NP_180536.1	*A. thaliana*
UGT73B1	NP_567955.1	*A. thaliana*
UGT73C6	NP_181217.1	*A. thaliana*
UGT74F2	OAP07463.1	*A. thaliana*
UGT75C1	AAL69494.1	*A. thaliana*
UGT71B2	NP_188813.1	*A. thaliana*
UGT78D2	NP_197207.1	*A. thaliana*
UGT89C1	Q9LNE6.1	*A. thaliana*
UGT74AN1	AXF50399.1	*Asclepias curassavica*
UGT94B1	Q5NTH0.1	*Bellis perennis*
UGT71F1	AAS94330.1	*Beta vulgaris*
UGT708G1	BBA18062.1	*Citrus japonica*
CmF7G2"RT	Q8GVE3.2	*Citrus maxima*
UGT708G2	BBA18063.1	*Citrus unshiu*
UGT78K6	4REL_A	*Clitoria ternatea*
UGT709G1	APU54677.1	*Crocus sativus*
F7GAT	ANC70234.1	*Erigeron breviscapus*
UGT708C1	BAP90360.1	*Fagopyrum esculentum*
FiF3GT	AAD21086.1	*Forsythia x intermedia*
UGT71A18	BAI65912.1	*F. x intermedia*
UGT73B4	NP_001354361.1	*Glycine max*
GmF3G2"GT	BAR88077.1	*G. max*
GmF3G6"RT	BAN91401.1	*G. max*
UGT78K1	ADC96620.1	*G. max*
UGT88E3.	NP_001235161.1	*G. max*
UGT73P12	BBN60804.1	*G. uralensis*
VhA5GT	Q9ZR25.1	*Glandularia x hybrida*
GeIF7GT	BAC78438.1	*Glycyrrhiza echinata*
UGT73F17	AXS75258.1	*Glycyrrhiza uralensis*
UGT72B11	ACB56923.1	*Hieracium pilosella*
HvF3GT	P14726.1	*Hordeum vulgare*
UGT79G16.	Q53UH5.1	*Ipomoea purpurea*
UGT71B5b	QTI0875.1	*Isatis indigotica*
UGT88D2	BAE48240.1	*Linaria vulgaris*
UGT74S1	AGD95005	*Linum usitatissimum*
UGT73A10	BAG80536.1	*Lycium barbarum*

UGT71A12	BAF96585.1	*Lycium chinense*
UGT71A13	ABL85473.1	*Maclura pomifera*
UGT75L4	ABL85474.1	*M. pomifera*
UGT88A4	ABL85471.2	*M. pomifera*
UGT71G1	XP_003615613.1	*Medicago truncatula*
UGT78G1	A6XNC6.1	*M. truncatula*
UGT88F1	NP_001315652.1	*Malus domestica*
MiCGTb	AMM73095.1	*Mangifera indica*
Ns3RT	BAC10994.1	*Nierembergia sp. NB17*
OcUGT1	AWD73588.1	*Ornithogalum longebracteatum*
UGT71A16	ACZ44836.1	*Pyrus communis*
UGT71K2	ACZ44837.1	*P. communis*
UGT88F2	ACZ44838.1	*P. communis*
PfF3GT	BAA19659.1	*P. frutescens*
PhA5GT	BAA89009.1	*P. x hybrida*
PhF3GT	BAA89008.1	*P. x hybrida*
PfA5GT	Q9ZR27.1	*Perilla frutescens*
PhA3G6"RT	CAA50376.1	*Petunia x hybrida*
PtUGT1	BBK15460.1	*Polygala tenuifolia*
RhA53GT	Q4R1I9.1	*Rosa hybrid cultivar*
SbB7GAT	Q76MR7.1	*Scutellaria baicalensis*
SbF7GT	BAA83484.1	*S. baicalensis*
UGT78B4	QBL54224.1	*S. baicalensis*
UGT94D1	BAF99027.1	*Sesamum indicum*
UGT71A9	XP_011100453.1	*S. indicum*
UGT71E1	Q6VAB2.1	*Stevia rebaudiana*
UGT74G1	AAR06920.1	*S. rebaudiana*
UGT71A8	BAF96581.1	*Sesamum alatum*
UGT71A10	BAF96583.1	*Sesamum radiatum*
TcCGT1	QCZ42162.1	*Trollius chinensis*
UGT74M1	ABK76266.1	*Vaccaria hispanica*
UGT73A16	ACO44747.1	*Withania somnifera*

Supplementary Table 4. ^{1}H-NMR, ^{13}C-NMR spectra of monoglycoside products.

	(+)-Lariciresinol-4-*O*-*β*-D-Glucoside		(+)-Lariciresinol-4′-*O*-*β*-D-Glucoside	
	δH	δC	δH	δC
1		134.3		132.1
2	6.89 (d, J = 1.8 Hz)	109.2	6.79 (d, J = 1.8 Hz)	112
3		149.5		149.4
4		145.7		145.9
5	7.09 (d, J = 8.2 Hz)	114.6	6.71 (d, J = 8.0 Hz)	114.8
6	6.74 (brs)	120.9	6.64 (dd, J = 8.0, 1.8 Hz)	120.7
7	2.98 (dd, J = 13.4, 4.8 Hz)	32.3	2.90 (dd, J = 13.5, 5.0 Hz)	32.2
	2.55 (dd, J = 13.4, 11.4 Hz)		2.50 (dd, J = 13.4, 11.2 Hz)	
8	2.72 - 2.77 (m)	42.4	2.68 - 2.74 (m)	42.4
9	3.99 (dd, J = 8.3, 6.5 Hz)	72	4.00 (dd, J = 8.3, 6.7 Hz)	72.3
	3.70 (dd, J = 8.4, 6.0 Hz)		3.74 (dd, J = 8.3, 6.4 Hz)	
3-OCH_3	3.84 (s)	55	3.83 (s)	55
1′		135.7		138.1
2′	6.90 (d, J = 1.8 Hz)	112.9	6.98 (d, J = 2.0 Hz)	109.9
3′		147.6		147.6
4′		145		144.4
5′	6.77 – 6.78 (m)	116.9	7.13 (d, J = 8.4 Hz)	116.5
6′	6.75 – 6.76 (m)	118.4	6.88 (dd, J = 8.4, 2.0 Hz)	118.2
7′	4.75 (d, J = 6.9 Hz)	82.6	4.83 (d, J = 6.4 Hz)	82.4
8′	2.35 - 2.40 (m)	52.7	2.32 - 2.37 (m)	52.7
9′	3.81 - 3.86 (m)	59	3.84 - 3.88 (m)	59.1
	3.62 - 3.65 (m)		3.66 - 3.68 (m)	
3′-OCH_3	3.85 (s)	55.3	3.86 (s)	55.3
Glc-1	4.87 (d, J = 7.6 Hz)	101.6	4.88 (d, J = 7.6 Hz)	101.5
Glc-2	3.43 - 3.49 (m)	73.5	3.44 - 3.48 (m)	73.5
Glc-3	3.57 - 3.60 (m)	76.4	3.57 - 3.60 (m)	76.4
Glc-4	3.38 - 3.40 (m)	70	3.36 - 3.40 (m)	70
Glc-5	3.66 - 3.68 (m)	76.8	3.63 - 3.66 (m)	76.8
Glc-6	3.85 - 3.88 (m)	61.1	3.84 - 3.88 (m)	61.1
	3.69 - 3.71 (m)		3.68 - 3.70 (m)	

Supplementary Table 5. List of primers used in the study.

Primers	Squences
IiUGT1F	GAGGGATCCATGAAGATTACAAGACCACA
IiUGT1R	GAGGCGGCCGCCTAGGCACCACGTGCCATTC
IiUGT2F	GAGCATATGGCGAAAGAAACAGAGCTCA
IiUGT2R	GAGGTCGACTCAGCTCGAACCATCGTCCA
IiUGT3F	GAGGGATCCATGCAAATCACAAAACCACA
IiUGT3R	GAGGCGGCCGCCTAAGCACCACGTGCCAAGT
IiUGT4F	GAGGGATCCATGAAGACATCGGAGCTAAT
IiUGT4R	GAGGCGGCCGCTCAAAAGTGATCCCCAAGAA
IiUGT5F	GAGGGATCCATGGAGCAGCCTCACGCGCT
IiUGT5R	GAGGTCGACCTATATTATTTGCGAATCAC
IiUGT6F	GAGGGATCCATGGAAAAGCAAAACGCAAT
IiUGT6R	GAGGTCGACTCATTTTGGGCTCCACGATT
IiUGT7F	GAGGCTAGCATGGGGAATCAAGAGATCATC
IiUGT7R	GAGGTCGACCTAAAAGTCCTCGCCGACCA
IiUGT8F	GAGGGATCCATGCAGAATACAAAACCTCA
IiUGT8R	GAGGCGGCCGCTTAGGCACCACGTGCCATGC
IiUGT9F	GAGGGATCCATGGAAAATCAAGAAGCTAT
IiUGT9R	GAGGTCGACTCATTTTGAGCTCCACGACT
IiUGT71B5aF	GAGGGATCCATGAAGATCGAGCTCGTGTT
IiUGT71B5aR	GAGGCGGCCGCCTAGACCACAACATTCTCGAT
138Actin-F	CCAGTGGTCGTACAACCGGTA
138Actin-R	TAGTTCTTTTCGATGGAGGAGCTG
IiUGT1RT-F	ATCAAAGGGTAAATGGACTG
IiUGT1RT-R	TCAGGTCTTGTCAAATGC
IiUGT4RT-4F	TGCTCTTCTCAAGTTCATTG
IiUGT4RT-4R	TGTACCTATATGCAATGTTTCTT
IiUGT71B5aRT-F	TTATCCTGTTGAATGCCTTC
IiUGT71B5aRT-R	AATTATCCGATCCGCTTTCA
IiUGT1I-1F	gatactagttctagagagcttgagggatacggcagagatg
IiUGT1I-1R	cggggaaattcgagctgtgactgcaagtggcgctg
IiUGT1I-2F	tcgagggtacccgggtgagggatacggcagagatg
IiUGT1I-2R	acgggggactctagaggtgactgcaagtggcgctg
IiUGT4I2-1F	gatactagttctagagagctgcacgctgctcttctcaagtt
IiUGT4I2-1R	cggggaaattcgagcttgcctaaaacatgtacctatatgcaatg
IiUGT4I2-2F	tcgagggtacccggggcacgctgctcttctcaag
IiUGT4I2-2R	acgggggactctagaggcctaaaacatgtacctatatg
IiUGT71B5aI-1F	gatactagttctagagagctgacgtgatcgagaatgttgtg
IiUGT71B5aI-1R	cggggaaattcgagctaggtctgaaccatctcaag
IiUGT71B5aI-2F	tcgagggtacccggggacgtgatcgagaatgttgtg
IiUGT71B5aI-2R	acgggggactctagagaggtctgaaccatctcaag

IiUGT1SCF	GCTTCTGCAGGGGCCCGGGGATGAAGATTACAAGACCACA
IiUGT1SCR	GGATCCACTAGTATTTAAATGGGCACCACGTGCCATTCTG
IiUGT4SCF	GCTTCTGCAGGGGCCCGGGGATGAAACAGGAGCTGGTTTTC
IiUGT4SCR	GGATCCACTAGTATTTAAATGAGAGATATTCGAAGTGACATC
IiUGT71B5aSCF	GCTTCTGCAGGGGCCCGGGGATGAAGATCGAGCTCGTGTTC
IiUGT71B5aSCR	GGATCCACTAGTATTTAAATGGACCACAACATTCTCGATCAC
RolB-F	CGAGGGGATCCGATTTGCTT
RolB-R	GACGCCCTCCTCGCCTTCCT
RolC-F	TCGCCATGCCTCACCAACTCAC
RolC-R	CCTTGATCGAGCCGGGTGAGAA
HPT-F	TACACAGCCATCGGTCCAGA
HPT-R	TTAGCGAGAGCCTGACCTATTG
224-35SF	GACGCACAATCCCACTATCC
NOS Ter SeqR	ATCATCGCAAGACCGGCAACAG
pZH02-F	GATAAAGAGTACCCACTGTATA
pZH02-R	caaccatgaacattaaagtg
SAIL_LB1	ggataaatagccttgcttcc
NOS Ter SeqR	ATCATCGCAAGACCGGCAACAG
IiUGT4F151A_F	cacctccaacgctacgGCTctcgggttg
IiUGT4F151A_R	GCcgtagcgttggaggtgtaaaacatgt
IiUGT4G14A_F	accatcacctggtgacGCCcacatcaga
IiUGT4G14A_R	GCgtcaccaggtgatggtatgaaaac
IiUGT4H373A_F	ccgaggatttgtgtcgGCCtgtggttgga
IiUGT4H373A_R	GCcgacacaaatcctcggacagcagggt
IiUGT4A207F_F	agtggctaccgattgcgGCTtcacaagtg
IiUGT4A207F_R	GCcgcaatcggtagccactccttgtttaa
IiUGT4D124A_F	ctggaatcgtggtggacGCGttctgcacg
IiUGT4D124A_R	GCgtccaccacgattccagcgagtcttga
IiUGT4N85D_F	tgatgacgccaaacccGACttcctctc
IiUGT4N85D_R	Cgggtttggcgtcatcagagtttggtt
IiUGT4N377A-F	gtcgcactgtggttggGCCtcgacactgg

IiUGT4N377A-R	GCccaaccacagtgcgacacaaatcctcg
IiUGT4Q398A_F	gccactctatgccgagGCAcaagttaacg
IiUGT4Q398A_R	GCctcggcatagagtggccacgtggcta
IiUGT4S196A_F	gttaagtgtttacccGCTgtgatgtt
IiUGT4S196A_R	Cgggtaaacacttaaccggcaaagga
IiUGT4S289A_F	gttcctctgttttggaGCCatgggaggtt
IiUGT4S289A_R	GCtccaaaacagaggaacacaacggttt
IiUGT4S289W_F	gttcctctgttttggaTGGatgggaggttt
IiUGT4S289W_R	CCAtccaaaacagaggaacacaacggttt
IiUGT4S378A-F	gcactgtggttggaacGCGacactggag
IiUGT4S378A-R	Cgttccaaccacagtgcgacacaaatc
IiUGT4W376A_F	gtgtcgcactgtggtGCGaactcgacac
IiUGT4W376A_R	GCaccacagtgcgacacaaatcctcgg
IiUGT4Y395F-F	agccacgtggccactcTTTgccgagcaac
IiUGT4Y395F-R	Aagagtggccacgtggctatcggaacacc

Supplementary Table 6. Mass spectrometry condition parameters of multi-target ingredients in *I. indigotica* Fort.

Compounds	Molecular formulas	Rt (min)	$[M\text{-}H]^-$	Negative ion mode Major Framents
Coniferyl alcohol	$C_{10}H_{12}O_3$	1.95	179.07	179.07,146.21,164.04
Coniferin	$C_{16}H_{22}O_8$	1.29	341.34	341.34,179.01
Pinoresinol	$C_{20}H_{22}O_6$	3.32	357.38	357.38,342.10,150.81
Pinoresinol diglucoside	$C_{32}H_{42}O_{16}$	1.78	682.66	680.90,519.02
Lariciresinol	$C_{20}H_{24}O_6$	2.84	360.4	359.10,328.90
(+)-Lariciresinol-4-*O*-*β*-D-glucoside	$C_{26}H_{34}O_{11}$	2.07	522.54	521.20,359.00,329.10
(+)-Lariciresinol-4'-*O*-*β*-D-glucoside	$C_{26}H_{34}O_{11}$	2.17	523.54	521.20,359.00,329.10
Clemastanin B	$C_{32}H_{44}O_{16}$	1.58	684.68	683.20,521.10,359.00
Secoisolariciresinol	$C_{20}H_{26}O_6$	2.71	362.41	361.14,179.91,164.92
Secoisolariciresinol diglucoside	$C_{32}H_{46}O_{16}$	2.11	686.69	684.94,523.24
Matairesinol	$C_{20}H_{22}O_6$	3.58	358.38	342.12,137.02,122.01
